# The efficacy and safety of endoscopic surgery combined with platelet-rich plasma for lumbar disk herniation: a systematic review and meta-analysis

**DOI:** 10.3389/fmed.2025.1697117

**Published:** 2025-12-05

**Authors:** Guimei Guo, Yu Cheng, Xinyue Yu, Wensi Ouyang, Changwei Zhao

**Affiliations:** 1 Changchun University of Chinese Medicine, Changchun, China; 2 The Affiliated Hospital to Changchun University of Chinese Medicine, Changchun, China; 3 Zigong Chinese Medicine Hospital, Zigong, China; 4 Hunan University of Chinese Medicine, Changsha, China

**Keywords:** endoscopic surgery, platelet-rich plasma, lumbar disk herniation, meta-analysis, systematic review

## Abstract

**Objective:**

Lumbar disk herniation (LDH) is a common spinal disorder with an increasing annual incidence, significantly impairing patients’ quality of life. In recent years, platelet-rich plasma (PRP) has emerged as a viable biologically based treatment alternative in clinical practice. The present study aimed to conduct a thorough review and meta-analysis to systematically assess the safety and effectiveness of endoscopic surgery combined with PRP for the treatment of LDH.

**Methods:**

A comprehensive search was conducted across eight databases from their inception until October 2025 to identify relevant articles assessing the efficacy of PRP therapy for LDH. Two independent reviewers carefully reviewed and selected studies using predefined inclusion and exclusion criteria. Furthermore, they assessed the eligible literature’s methodological quality. Visual analog scale (VAS) scores for back and leg pain, Japanese Orthopedic Association (JOA) scores, Oswestry Disability Index (ODI) scores, disk height, treatment-related complications, and recurrence were among the outcome measures examined. Stata version 17.0 and Review Manager version 5.4.1 were used for statistical analysis. In addition, the Grading of Recommendations, Assessment, Development, and Evaluation (GRADE) approach was used to assess the quality of evidence for each outcome.

**Results:**

This comprehensive analysis included 12 studies comprising 960 patients diagnosed with LDH. The aggregated results demonstrated significant reductions in both back and leg VAS scores across all evaluated time points. For back pain, VAS scores decreased significantly at 3 months (MD = −0.58, 95% CI: −0.84 to −0.31, *p* < 0.0001), 6 months (MD = −0.32, 95% CI: −0.48 to −0.16, *p* = 0.0001), ≥12 months (MD = −0.40, 95% CI: −0.51 to −0.28, *p* < 0.00001) and at last follow-up (MD = −0.23, 95% CI: −0.36 to −0.10, *p* = 0.0005; very low certainty). Similarly, leg VAS scores were significantly reduced at 3 months (MD = −0.38, 95% CI: −0.52 to −0.24, *p* < 0.00001); 6 months (MD = −0.53, 95% CI: −0.67 to −0.38, *p* < 0.00001); ≥12 months (MD = −0.38, 95% CI: −0.62 to −0.14, *p* = 0.002); and at last follow-up (MD = −0.35, 95% CI: −0.60 to −0.10, *p* = 0.007; very low certainty). JOA scores increased significantly at all time points: 3 months (MD = 1.91, 95% CI: 0.65 to 3.18, *p* = 0.003), 6 months (MD = 0.97, 95% CI: 0.44 to 1.50, *p* = 0.0003), ≥12 months (MD = 1.60, 95% CI: 0.37 to 2.84, *p* = 0.01), and at last follow-up (MD = 1.26, 95% CI: 0.31 to 2.21, *p* = 0.009; very low certainty). ODI scores also showed significant improvements at 3 months (MD = −2.64, 95% CI: −4.19 to −1.08, *p* = 0.0009); 6 months (MD = −2.05, 95% CI: −2.95 to −1.14, *p* < 0.00001); ≥12 months (MD = −2.72, 95% CI: −5.22 to −0.22, *p* = 0.03); and at last follow-up (MD = −1.53, 95% CI: −2.45 to −0.61, *p* = 0.001; very low certainty). There was a significant difference in disk height (MD = 0.74, 95% Cl: 0.52 to 0.97, *p* < 0.00001; low certainty) and recurrence (RR = 0.27, 95% Cl: 0.12 to 0.60, *p* = 0.001; low certainty). The analysis revealed no statistically significant difference in the incidence of complication (RR = 0.81, 95% Cl: 0.38 to 1.73, *p* = 0.58; very low certainty).

**Conclusion:**

The synthesized findings suggest that endoscopic surgery combined with PRP treatment alleviates clinical symptoms and improves the quality of life in LDH patients. However, due to methodological limitations and potential heterogeneity across studies, higher-quality research is required to substantiate its efficacy and safety for LDH.

## Introduction

Lumbar disk herniation (LDH) is a common spinal disorder characterized by the gradual degeneration of intervertebral disks, leading to annulus fibrosus rupture and subsequent displacement of the nucleus pulposus (1–3). This pathological progression results in mechanical nerve root compression and inflammatory stimulation, clinically manifesting as lower back pain, radiating pain in the lower limbs, and sensory impairments. These symptoms considerably reduce daily functioning, adversely impacting patients’ occupational performance and quality of life (4, 5). Recent socioeconomic advances and shifting lifestyle habits have contributed to the rising incidence of LDH, increasingly affecting younger populations and generating significant economic implications. Consequently, an urgent clinical demand exists for effective and safe LDH treatments.

The LDH is recognized as a multifactorial degenerative disorder, although its precise etiopathogenesis remains unclear. Recent studies show associations with degenerative processes, mechanical stress injuries, and immune-inflammatory responses (6, 7). Clinically, oral medications, physiotherapy, and exercise therapy can alleviate some symptoms but fail to promote recovery of the lumbar disk (8–10). Currently, no effective treatments exist to slow or reverse lumbar disk degeneration, making surgery an inevitable option for advanced LDH. Therefore, designing novel treatment plans that alleviate clinical symptoms and postpone disk degeneration is imperative.

Platelet-rich plasma (PRP) therapy has recently demonstrated significant regenerative potential in cartilage, tendons, ligaments, and skin and has been extensively studied across multiple medical fields (11–13). The PRP is a concentration of platelets derived from the patient’s blood through high-speed centrifugation. It is rich in growth factors, chemokines, and fibrin, which help regulate the local microenvironment within lumbar intervertebral disks (14, 15). Researchers suggest that by releasing various bioactive factors, PRP reduces inflammatory cell production and promotes nucleus pulposus cell proliferation (16–18). Furthermore, PRP is easy to prepare, widely available, and highly safe. Thus, PRP holds promise as a biological treatment for LDH. The purpose of this meta-analysis was to thoroughly evaluate the clinical efficacy of endoscopic surgery combined with PRP for LDH to offer solid, evidence-based recommendations for well-informed clinical decision-making.

## Methods and materials

### Protocol register

The Preferred Reporting Items for Systematic Reviews and Meta-Analyses criteria were strictly followed in the conduct of this systematic review and meta-analysis, which was prospectively filed with PROSPERO (Registration no. CRD42024552638) (19, 20).

### Search strategy

At separate times, two researchers searched eight electronic databases: China Science and Technology Journal Database, WanFang Database, Chinese Biomedical Literature Database, Web of Science, EMBASE, Cochrane Library, PubMed, and Chinese National Knowledge Infrastructure. The database search was available worldwide and did not restrict the results based on language or location from the beginning until 1 October 2025. We customized each database by combining appropriate medical subject headings with free-text phrases. We systematically examined pertinent reviews, gray literature, and reference lists of the identified papers to uncover more relevant studies. Comprehensive search strategies for each database are found in the Supplementary material.

## Eligibility criteria

### Inclusion criteria

(a) Participants: All participants had a clinical and radiological diagnosis of LDH. There were no restrictions regarding age, gender, race, nationality, geographic region, or symptom duration.
(b) Interventions: Studies assessing the efficacy of endoscopic surgery combined with PRP for LDH treatment were included, irrespective of PRP preparation or application methods.
(c) Comparisons: Endoscopic surgical therapy without PRP injection served as the control treatment.
(d) Outcome measures: The outcomes included improvements in visual analog scale (VAS) score (pain rating scales ranging from 0 to 10 for both back and leg pain, with higher values indicating higher pain intensity), Oswestry Disability Index (ODI) score (activity function scales rated from 0 to 100, with lower values indicating improvement), and Japan Orthopedic Association (JOA) score (functional status questionnaires rated from 0 to 29, with higher values indicating better function). Additionally, intervertebral disk height was measured radiographically, with higher values indicating structural improvement. Relevant complications and recurrences were also recorded.
(e) Study types: Cohort studies, case–control studies, and randomized controlled trials (RCTs) published in either Chinese or English were among the studies deemed appropriate for inclusion.

### Exclusion criteria

(a) Publications reporting overlapping patient populations or duplicate studies.
(b) Technical notes, case studies, editorials, conference abstracts, review articles, animal studies, basic experimental investigations, and narrative reviews.
(c) Studies lacking sufficient or accessible data.

### Literature screening and data extraction

Two reviewers independently assessed study eligibility based on the predefined inclusion criteria. Any disagreements were resolved through discussion or, if required, consultation with a third reviewer. Key information, including study authorship, publication year, sample characteristics (size, gender distribution, and mean age), intervention details, treatment duration, PRP preparation and administration techniques, follow-up period, and reported outcomes, was captured by two authors during the independent data extraction process. Moreover, outcome-specific data extraction for synthesis was independently performed by two reviewers.

### Risk of bias of individual studies

The quality of selected research was evaluated separately by two authors. For RCTs, quality assessment was performed using the Cochrane Risk of Bias assessment tool, version 2 (RoB-2) (21). Non-randomized controlled trials were evaluated using the Cochrane’s Risk of Bias In Non-Randomized Studies of Interventions (ROBINS-I) (22).

### Quality of evidence assessment

To determine the level of certainty in the evidence, two researchers worked separately and used the Grades of Recommendation Assessment, Development, and Evaluation (GRADE) technique (23). This assessment includes the risk of bias, inconsistency, indirectness, imprecision, and publication bias (24). A third reviewer was brought in to discuss and arbitrate any disagreements in judgment. Transparent documentation was maintained for all decisions about grading modifications.

### Statistical analysis

Data analysis was conducted using Review Manager (version 5.4.1) and Stata (version 17.0). Risk ratios (RR) were calculated for categorical variables, and mean differences (MD) were computed for continuous variables. The results were presented with a 95% confidence interval (CI) to demonstrate the comparative effects of the interventions relative to the controls. The *I^2^* statistic and the chi-square test were used to assess heterogeneity. When heterogeneity was minimal (*I^2^* < 50%), a fixed-effects model was used; when heterogeneity was substantial, a random-effects model was used. If *I^2^* exceeded 50%, a subgroup analysis was conducted based on the study design and age to identify the source of heterogeneity. Sensitivity analyses were performed by sequentially omitting individual studies to assess the stability of the results. Using Egger’s statistical test and visual examination of funnel plots, publication bias was investigated.

## Results

### Search selection

**Figure 1:**
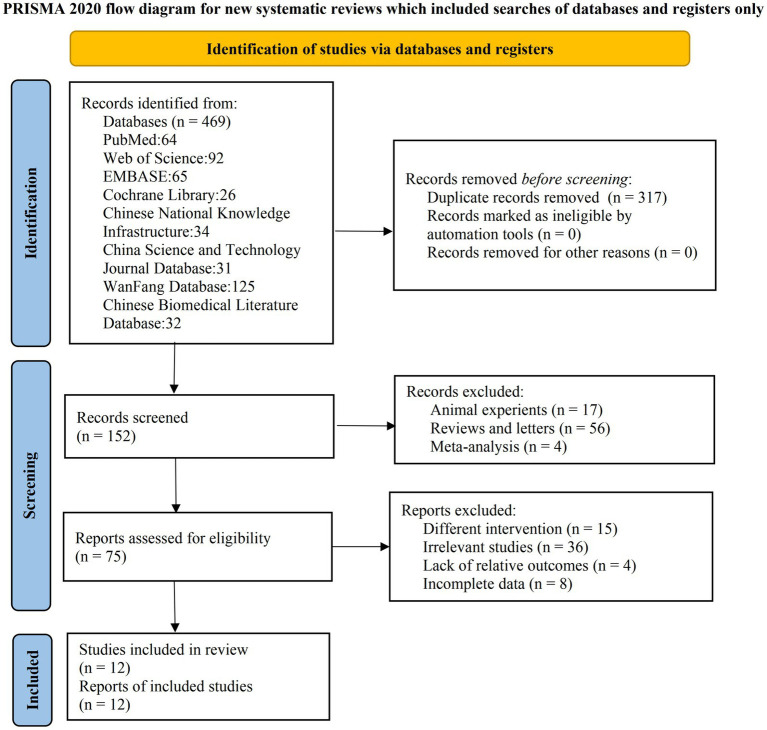
Literature screening process of the meta-analysis. From: Page MJ, McKenzie JE, Bossuyt PM, Boutron I, Hoffmann TC, Mulrow CD, et al. The PRISMA 2020 statement: an updated guideline for reporting systematic reviews. BMJ 2021;372:n71. doi: 10.1136/bmj.n71.

The preliminary database search identified 469 articles on PRP treatment for LDH. After removing 317 duplicate records, 152 records remained for title and abstract screening, resulting in the exclusion of 77 studies. These were eliminated after a full-text review of 63 more papers unsuitable for the predetermined inclusion and exclusion criteria. Finally, the 12 papers (2, 25–35) satisfied the requirements for inclusion in the meta-analysis (Figure 1).

### Baseline characteristics

The 12 included studies (2, 25–35) involved a total of 960 LDH patients: 484 participants assigned to the control groups and 476 to the PRP intervention groups. These studies were published between 2020 and 2024. Participant ages varied from 32.35 years to 49.12 years in the control groups and 32.55 years to 48.36 years in the PRP groups. Follow-up duration varied from 6 months to approximately 28.03 months. Each included study explicitly outlined inclusion and exclusion criteria, demonstrating balanced baseline characteristics between the groups. Professional or governmental funding sources were acknowledged in seven studies (26–28, 30, 31, 33–35), whereas five studies (2, 25, 29, 32) did not disclose funding information. Although all studies described PRP preparation, the levels of procedural detail differed across studies. Detailed study characteristics are summarized in Tables 1–3.

**Table 1:** Basic characteristics of the 14 studies included in this review.

Inclusion studies	Study design	Sample size (male/female)	Patient age (years)	BMI (kg/m^2^)	Disease duration
Fu et al. (25)	Cohort study	T: 38 (21/17) C: 34 (19/15)	T: 37.15 ± 4.02°C: 38.32 ± 4.56	T: 21.05 ± 0.61°C: 20.91 ± 0.56	T: 10.85 ± 0.72 months C: 11.02 ± 0.59 months
Li et al. (26)	Case–control study	T: 30 (17/13) C: 30 (18/12)	T: 37.10 ± 11.0°C: 36.90 ± 10.7	T: 24.20 ± 2.6°C: 24.10 ± 1.8	T: 29.0 ± 7.9 months C: 28.7 ± 8.9 months
Li et al. (27)	Cohort study	T: 75 (39/36) C: 80 (46/34)	T: 43.61 ± 11.72°C: 44.25 ± 11.56	T: 24.22 ± 2.98°C: 24.41 ± 3.09	T: 21.87 ± 7.86 months C: 23.54 ± 8.40 months
Qi et al. (28)	Randomized controlled trial	T: 30 (16/14) C: 30 (18/12)	T: 44.20 ± 7.32°C: 43.30 ± 6.26	T: 25.89 ± 1.64°C: 26.54 ± 1.73	T: 5.98 ± 4.46 months C: 6.40 ± 5.21 months
Su (29)	Randomized controlled trial	T: 49 (28/21) C: 49 (26/23)	T: 46.85 ± 9.28°C: 46.18 ± 8.92	T: 23.74 ± 2.89°C: 23.56 ± 3.02	T: NA C: NA
Li et al. (30)	Case–control study	T: 29 (16/13) C: 29 (17/12)	T: 43.38 ± 14.01°C: 42.93 ± 10.37	T: 23.28 ± 3.61°C: 22.69 ± 3.54	T: 26.03 ± 3.54 months C: 25.72 ± 3.10 months
Li et al. (31)	Case–control study	T: 38 (30/8) C: 40 (32/8)	T: 32.55 ± 7.25°C: 32.35 ± 6.32	T: 25.51 ± 4.34°C: 24.97 ± 3.71	T: 23.26 ± 3.49 months C: 22.83 ± 3.95 months
Zhang et al. (2)	Case–control study	T: 50 (28/22) C: 48 (26/22)	T: 41.5 ± 9.8°C: 45.8 ± 11.0	T: 21.3 ± 3.1°C: 23.4 ± 2.9	T: 12.1 ± 10.1 months C: 15.3 ± 8.6 months
Lin et al. (32)	Case–control study	T: 36 (32/19) C: 37 (17/20)	T: 48.36 ± 9.53°C: 49.12 ± 8.72	T: 28.92 ± 3.21°C: 28.47 ± 2.91	T: 10.58 ± 4.91 months C: 10.06 ± 5.16 months
Jiang et al. (33)	Cohort study	T: 51 (21/15) C: 57 (33/24)	T: 48.1 ± 10.25°C: 45.9 ± 9.83	T: NA C: NA	T: NA C: NA
Lu et al. (34)	Case–control study	T: 30 (NA) C: 30 (NA)	T: NA C: NA	T: NA C: NA	T: NA C: NA
Du et al. (35)	Randomized controlled trial	T: 20 (11/9) C: 20 (10/10)	T: 47.80 ± 13.46°C: 45.85 ± 10.45	T: 22.43 ± 2.03°C: 23.22 ± 2.19	T: 18.40 ± 12.51 months C: 21.85 ± 13.36 months

C, control group; NA, not available; T, treatment group.

**Table 2:** Intervention characteristics of the included studies.

Inclusion studies	Treatment group	Control group	Lumbar level	Outcomes	Follow-up
Fu et al. (25)	PRP + percutaneous endoscopic interlaminar discectomy	Percutaneous endoscopic interlaminar discectomy	T: L3/L4 = 9, L4/L5 = 22, L5/S1 = 7°C: L3/L4 = 7, L4/L5 = 19, L5/S1 = 8	Back VAS scores JOA scores ODI scores Disc height Complication	12 months
Li et al. (26)	PRP + percutaneous endoscopic interlaminar discectomy	Percutaneous endoscopic interlaminar discectomy	T: L4/L5 = 15, L5/S1 = 15°C: L4/L5 = 18, L5/S1 = 12	Back VAS scores Leg VAS scores JOA scores ODI scores Recurrence	27.2 ± 2.0 months
Li et al. (27)	PRP + percutaneous endoscopic lumbar discectomy	Percutaneous endoscopic lumbar discectomy	T: L3/L4 = 10, L4/L5 = 36, L5/S1 = 29°C: L3/L4 = 12, L4/L5 = 40, L5/S1 = 28	Back VAS scores Leg VAS scores JOA scores ODI scores Recurrence	12 months
Qi et al. (28)	PRP + percutaneous endoscopic nucleotomy	Percutaneous endoscopic nucleotomy	T: L3/L4 = 5, L4/L5 = 14, L5/S1 = 11°C: L3/L4 = 4, L4/L5 = 13, L5/S1 = 13	Back VAS scores ODI scores	6 months
Su (29)	PRP + percutaneous endoscopic lumbar discectomy	Percutaneous endoscopic lumbar discectomy	T: L3/L4 = 4, L4/L5 = 35, L5/S1 = 10°C: L3/L4 = 5, L4/L5 = 32, L5/S1 = 12	Back VAS scores Leg VAS scores ODI scores Disc height	12 months
Li et al. (30)	PRP + percutaneous transforaminal endoscopic discectomy	Percutaneous transforaminal endoscopic discectomy	T: L4/L5 = 16, L5/S1 = 13°C: L4/L5 = 19, L5/S1 = 10	Back VAS scores Leg VAS scores JOA scores ODI scores Disc height Recurrence	T: 28.03 ± 2.69 months C: 27.86 ± 2.17 months
Li et al. (31)	PRP + percutaneous transforaminal endoscopic discectomy	Percutaneous transforaminal endoscopic discectomy	T: L4/L5 = 20, L5/S1 = 18°C: L4/L5 = 22, L5/S1 = 18	Back VAS scores JOA scores ODI scores Recurrence	28.02 ± 2.19 months
Zhang et al. (2)	PRP + percutaneous endoscopic lumbar discectomy	Percutaneous endoscopic lumbar discectomy	T: L3/L4 = 9, L4/L5 = 18, L5/S1 = 23°C: L3/L4 = 8, L4/L5 = 16, L5/S1 = 24	Back VAS scores Leg VAS scores JOA scores ODI scores Disc height Recurrence	18 months
Lin et al. (32)	PRP + percutaneous transforaminal endoscopic discectomy	Percutaneous transforaminal endoscopic discectomy	T: L3/L4 = 6, L4/L5 = 21, L5/S1 = 9°C: L3/L4 = 8, L4/L5 = 16, L5/S1 = 13	Back VAS scores ODI scores Complication	15.37 ± 1.92 months
Jiang et al. (33)	PRP + transforaminal endoscopic lumbar discectomy	Transforaminal endoscopic lumbar discectomy	T: L3/L4 = 6, L4/L5 = 33, L5/S1 = 12°C: L3/L4 = 7, L4/L5 = 30, L5/S1 = 20	Back VAS scores Leg VAS scores ODI scores Disc height Recurrence	12 months
Lu et al. (34)	PRP + percutaneous transforaminal endoscopic discectomy	Percutaneous transforaminal endoscopic discectomy	L3/L4 = 8, L4/L5 = 37, L5/S1 = 15	Back VAS scores JOA scores ODI scores	6 months
Du et al. (35)	PRP + percutaneous endoscopic lumbar discectomy	Percutaneous endoscopic lumbar discectomy	T: L3/L4 = 4, L4/L5 = 9, L5/S1 = 7°C: L3/L4 = 3, L4/L5 = 11, L5/S1 = 6	Back VAS scores JOA scores ODI scores Disc height Complication	24.2 ± 1.9 months

C, control group; JOA, Japanese Orthopedic Association; NA, not available; ODI, Oswestry Disability Index; PRP, platelet-rich plasma; T, treatment group; VAS, visual analog scale.

**Table 3:** PRP preparation techniques and application schemes in the studies.

Inclusion studies	Extracted blood volume	Preparation method	Centrifugation parameters	PRP dosage	Application scheme
Fu et al. (25)	60 mL	Centrifugation	Two centrifugations: -First at 2000 rpm (10 min) -Second at 2350 rpm (10 min)	4 mL	One injection
Li et al. (26)	36 mL	Centrifugation	Two centrifugations: -First at 2000 rpm (10.5 min) -Second at 2350 rpm (10.5 min)	4 mL	One injection
Li et al. (27)	36 mL	Centrifugation	Two centrifugations: -First at 2000 rpm (10.5 min) -Second at 2350 rpm (10.5 min)	4 mL	One injection
Qi et al. (28)	18 mL	Centrifugation	Two centrifugations: -First at 1495 rpm (10 min) -Second at 1978 rpm (10 min)	3 mL	One injection
Su (29)	50 mL	Centrifugation	Two centrifugations: -First at 2500 rpm (10 min) -Second at 2750 rpm (10 min)	4 mL	One injection
Li et al. (30)	36 mL	Centrifugation	Two centrifugations: -First at 2000 rpm (10.5 min) -Second at 2350 rpm (10.5 min)	4 mL	One injection
Li et al. (31)	36 mL	Centrifugation	Two centrifugations: -First at 2000 rpm (10.5 min) -Second at 2350 rpm (10.5 min)	4 mL	One injection
Zhang et al. (2)	30 mL	Centrifugation	Two centrifugations: NA	3.5–4 mL	One injection
Lin et al. (32)	60 mL	Centrifugation	Two centrifugations: -First at 200 g (10 min) -Second at 800 rpm (6 min)	5 mL	One injection
Jiang et al. (33)	NA	Centrifugation	Two centrifugations: -First at 2500 rpm (10 min) -Second at 2750 rpm (10 min)	4 mL	One injection
Lu et al. (34)	40 mL	Centrifugation	Two centrifugations: -First at 100 g (10 min) -Second at 400 rpm (10 min)	4 mL	One injection
Du et al. (35)	30 mL	Centrifugation	NA	3 mL	One injection

NA, Not available.

### Risk of bias assessment

**Figure 2:**
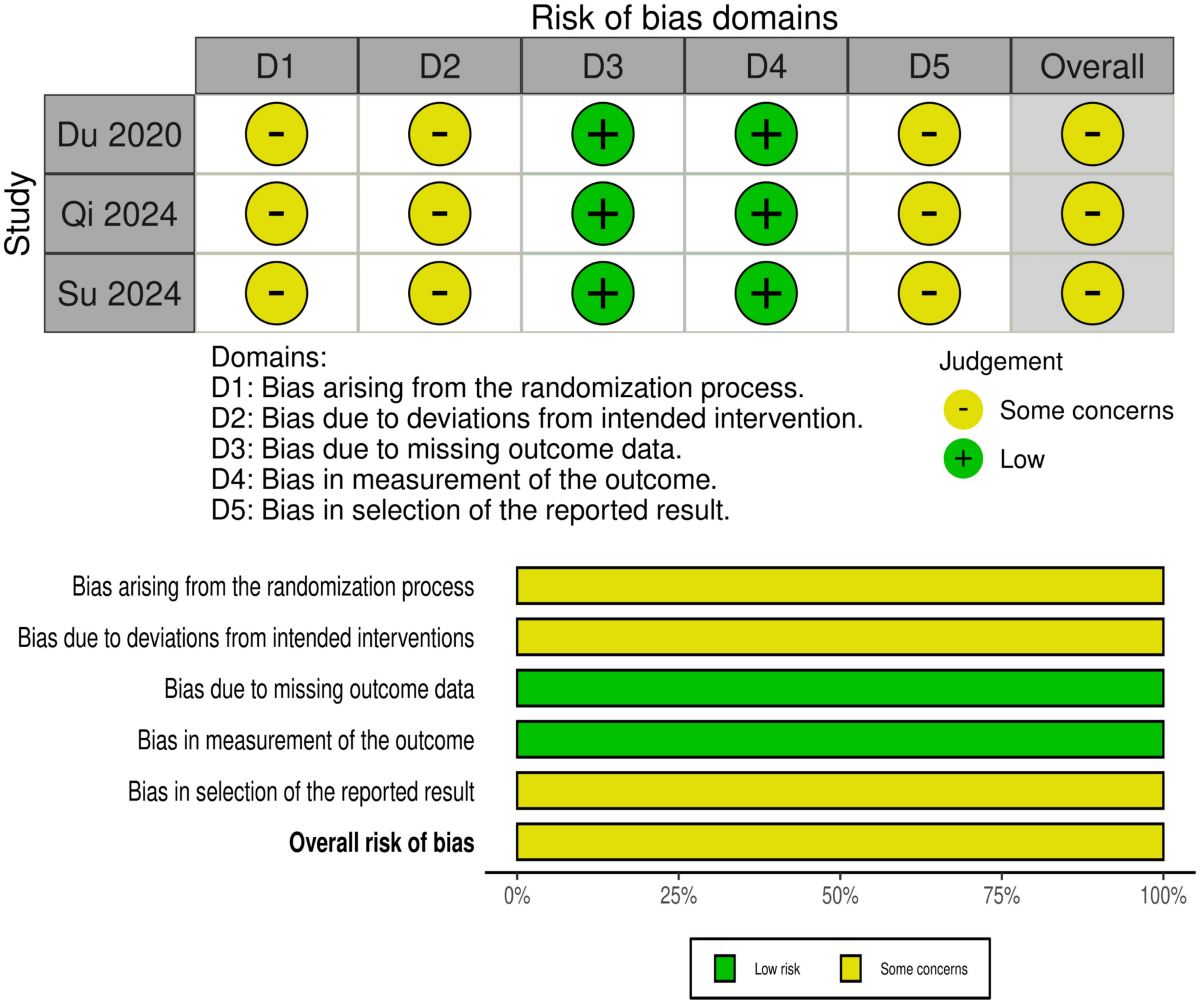
Assessment of risk of bias of randomized controlled trials with RoB-2.

**Figure 3:**
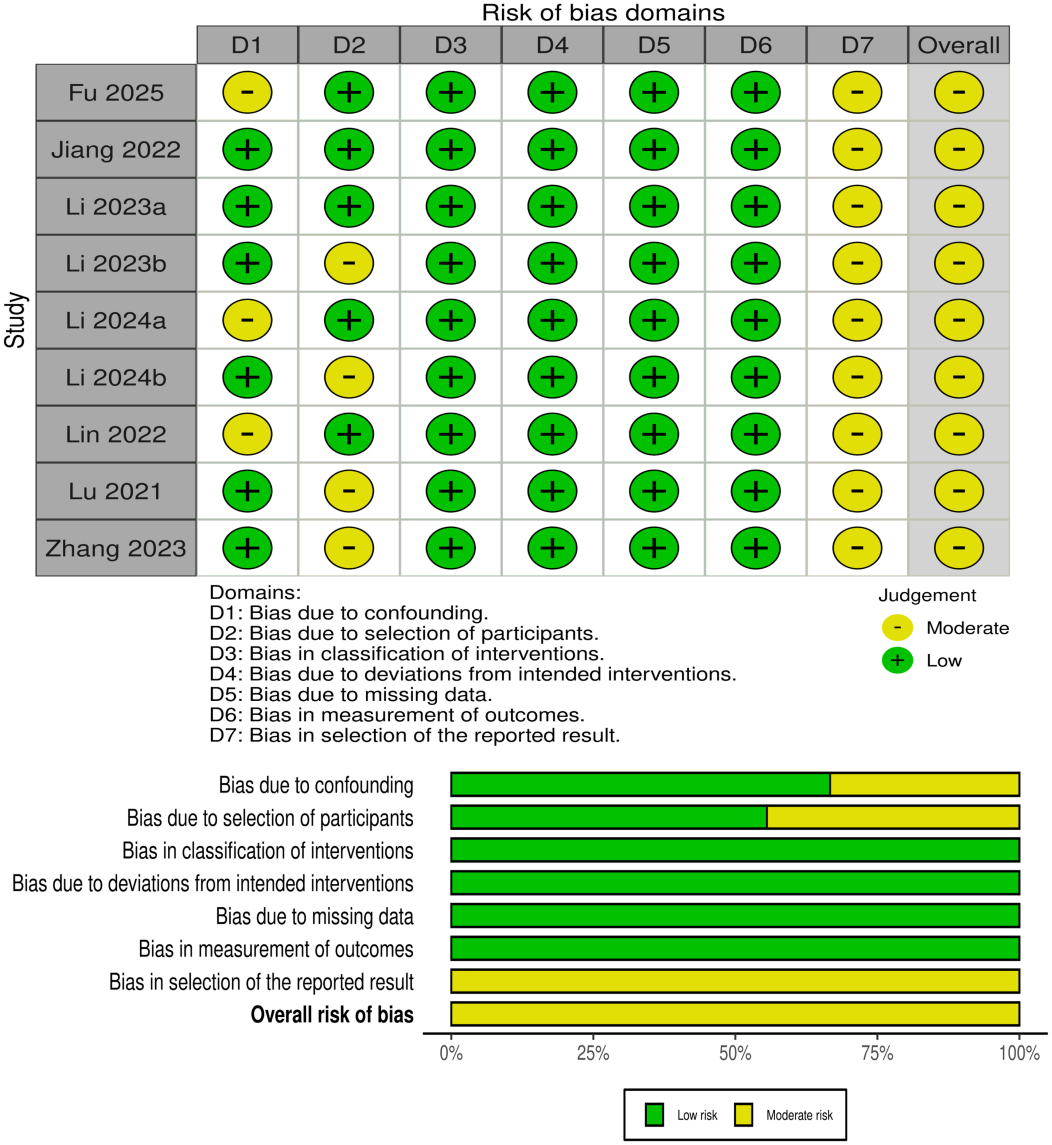
Assessment of risk of bias of non-randomized controlled trials with ROBINS-I.

Three RCTs (28, 29, 35) had some concerns regarding the randomization process (lack of allocation concealment description), deviation from the intervention (lack of blinding), and selective reporting of outcomes (Figure 2). Nine non-randomized controlled trials (2, 25–27, 30–34) demonstrated a moderate risk of bias primarily due to issues with confounding factors, participant selection, and selective reporting (Figure 3).

## Meta-analysis results

### Back VAS scores

**Figure 4:**
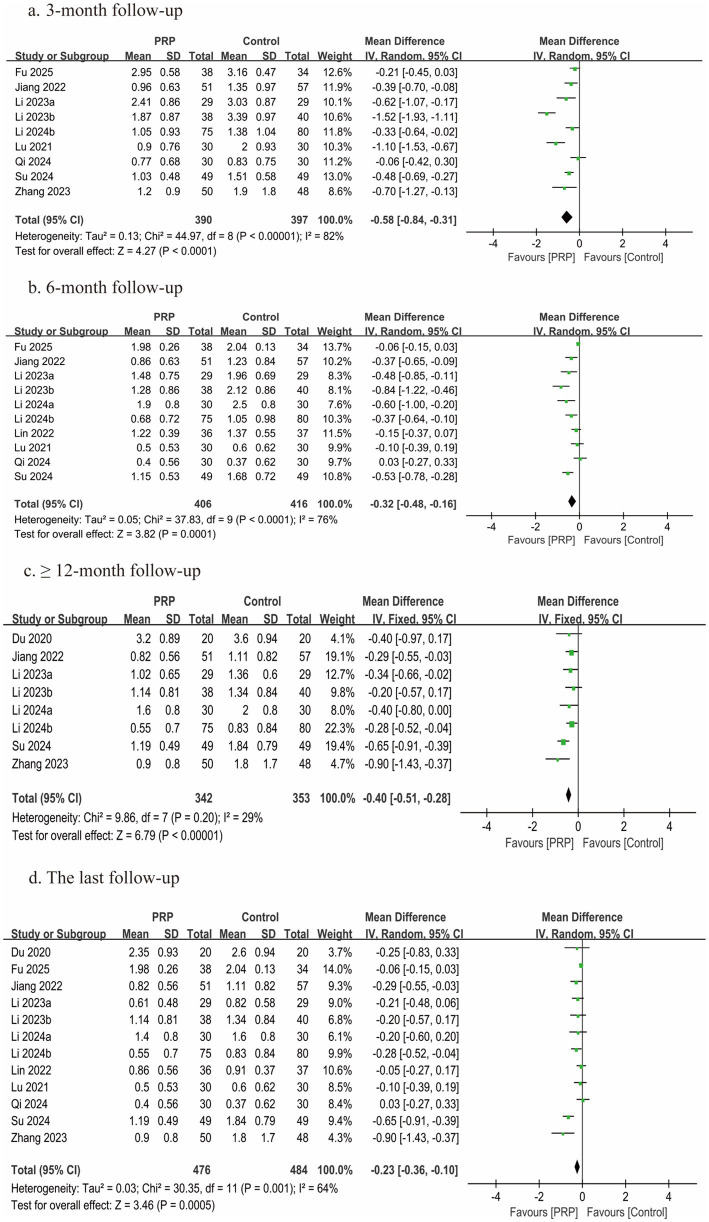
Forest plot of the meta-analysis comparing back VAS scores. **(a)** The duration of follow-up was 3 months, **(b)** the duration of follow-up was 6 months, **(c)** the duration of follow-up was longer than 12 months, and **(d)** the last follow-up.

Back VAS scores were reported in all 12 studies (2, 25–35) comprising 960 patients; each study was judged to have a moderate risk of bias. Data at 3 months were available from nine studies (2, 25, 27–31, 33, 34), data at 6 months were available from 10 studies (25–34), and data at ≥12 months were available from eight studies (2, 26, 27, 29–31, 33, 35). In all follow-up periods (3, 6, and ≥12 months) and at the final follow-up, statistically significant differences between the groups were found by the meta-analysis (3 months, *n* = 787, MD = −0.58, 95% CI: −0.84 to −0.31, *I^2^* = 82%, *p* < 0.0001; 6 months, *n* = 822, MD = −0.32, 95% CI: −0.48 to −0.16, *I^2^* = 76%, *p* = 0.0001; ≥12 months, *n* = 695, MD = −0.40, 95% CI: −0.51 to −0.28, *I^2^* = 29%, *p* < 0.00001; last follow-up, n = 960, MD = −0.23, 95% CI: −0.36 to −0.10, *I^2^* = 64%, *p* = 0.0005; very low certainty) (Figure 4).

Subgroup analyses were performed based on study design and patient age. The pooled results revealed no statistically significant impact on back VAS scores in either the RCT subgroup (*n* = 198, MD = −0.30, 95% CI: −0.78 to 0.19, *I^2^* = 82%, *p* = 0.23) or the subgroup of patients aged < 40 years (*n* = 210, MD = −0.07, 95% CI: −0.16 to 0.01, *I^2^* = 0%, *p* = 0.10; Table 4; Supplementary Figures S1, S2).

**Table 4:** Summary data and analyses.

Outcomes	Subgroups	Number of studies	Effect estimate (MD)	Heterogeneity		Group deference (*p*-value)
				*p*-value	*I*^2^ (%)	
Back VAS scores						
	RCT	3	−0.30 [−0.78 to 0.19]	0.003	82	0.65
	Non-RCT	9	−0.18 [−0.30 to −0.07]	0.07	44	
	< 40 years old	3	−0.07 [−0.16 to 0.01]	0.63	0	0.04
	≥ 40 years old	8	−0.29 [−0.48 to −0.11]	0.003	68	
Leg VAS scores						
	RCT	1	−0.81 [−1.08 to −0.54]	–	–	0.0006
	Non-RCT	5	−0.25 [−0.42 to −0.09]	0.34	12	
	< 40 years old	1	−0.20 [−0.63 to 0.23]	–	–	0.53
	≥ 40 years old	5	−0.37 [−0.65 to −0.08]	0.004	74	
JOA scores						
	RCT	1	1.20 [−0.23 to 2.63]	–	–	0.94
	Non-RCT	7	1.27 [0.23 to 2.31]	<0.00001	90	
	< 40 years old	3	0.63 [0.13 to 1.13]	0.86	0	0.17
	≥ 40 years old	4	2.08 [0.09 to 4.07]	<0.00001	93	
ODI scores						
	RCT	3	−1.73 [−4.90 to 1.45]	<0.00001	93	0.93
	Non-RCT	9	−1.57 [−2.83 to −0.31]	<0.00001	92	
	< 40 years old	3	−1.24 [−3.18 to 0.71]	0.12	54	0.55
	≥ 40 years old	8	−2.06 [−3.90 to −0.21]	<0.00001	94	

### Leg VAS scores

**Figure 5:**
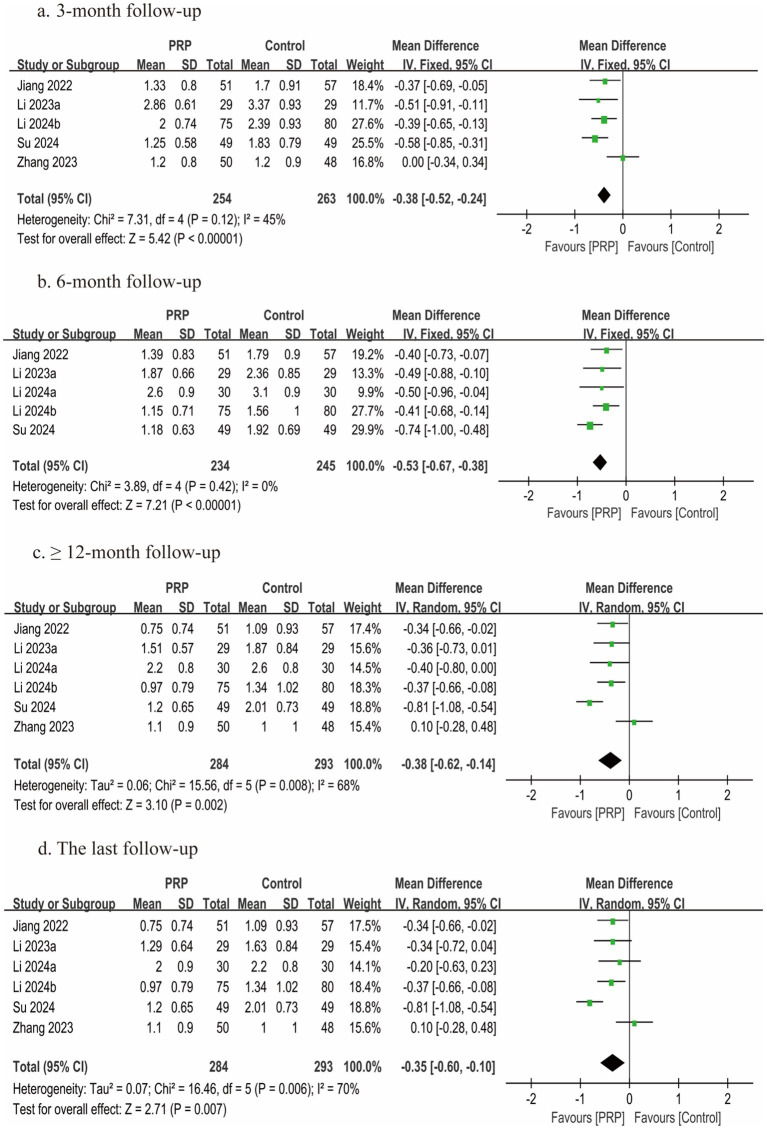
Forest plot of the meta-analysis comparing leg VAS scores. **(a)** The duration of follow-up was 3 months, **(b)** the duration of follow-up was 6 months, **(c)** the duration of follow-up was longer than 12 months, and **(d)** the last follow-up.

Six studies (2, 26, 27, 29, 30, 33) encompassing 577 patients provided leg VAS scores data; each study was judged to have a moderate risk of bias. Five studies (2, 27, 29, 30, 33) reported leg VAS scores at 3 months, five studies (26, 27, 29, 30, 33) reported leg VAS scores at 6 months, and six studies (2, 26, 27, 29, 30, 33) reported leg VAS scores at ≥12 months post-treatment. In all follow-up periods (3, 6, and ≥12 months) and at the final follow-up, statistically significant differences between groups were found by the meta-analysis (3 months, *n* = 517, MD = −0.38, 95% CI: −0.52 to −0.24, *I^2^* = 45%, *p* < 0.00001; 6 months, *n* = 479, MD = −0.53, 95% CI: −0.67 to −0.38, *I^2^* = 0%, *p* < 0.00001; ≥12 months, *n* = 577, MD = −0.38, 95% CI: −0.62 to −0.14, *I^2^* = 68%, *p* = 0.002; last follow-up, *n* = 577, MD = −0.35, 95% CI: −0.60 to −0.10, *I^2^* = 70%, *p* = 0.007; very low certainty) (Figure 5).

A meaningful pooled analysis was precluded for both the RCT subgroup and the subgroup of patients under 40 years of age, as each contained only a single eligible study (Table 4; Supplementary Figures S3, S4).

### JOA scores

**Figure 6:**
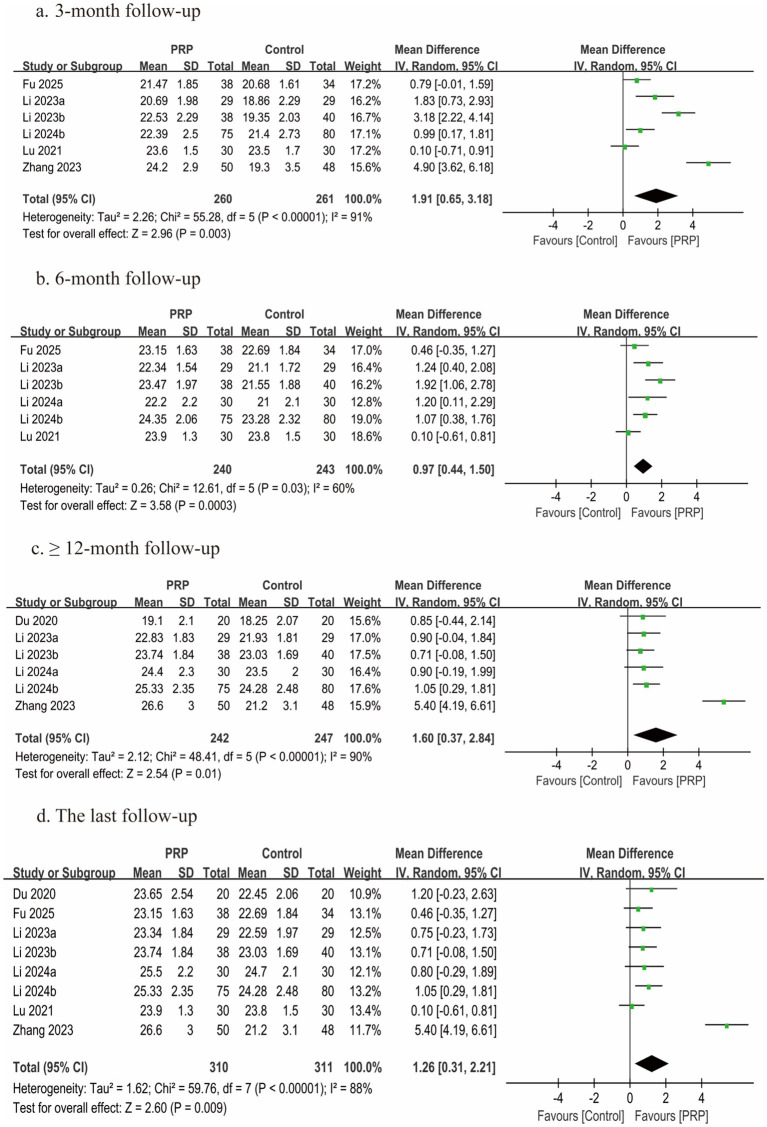
Forest plot of the meta-analysis comparing JOA scores. **(a)** The duration of follow-up was 3 months, **(b)** the duration of follow-up was 6 months, **(c)** the duration of follow-up was longer than 12 months, and **(d)** the last follow-up.

Eight studies (2, 25–27, 30, 31, 34, 35) involving 621 participants presented JOA scores; each study was classified as having a moderate risk of bias. Six studies (2, 25, 27, 30, 31, 34) reported JOA scores at 3 months, six studies (25–27, 30, 31, 34) reported JOA scores at 6 months, and six studies (2, 26, 27, 30, 31, 35) reported JOA scores at ≥12 months post-treatment. In all follow-up periods (3, 6, and ≥12 months) and at the final follow-up, statistically significant differences between the groups were found by the meta-analysis (3 months, *n* = 521, MD = 1.91, 95% CI: 0.65 to 3.18, *I^2^* = 91%, *p* = 0.003; 6 months, *n* = 483, MD = 0.97, 95% CI: 0.44 to 1.50, *I^2^* = 60%, *p* = 0.0003; ≥12 months, *n* = 489, MD = 1.60, 95% CI: 0.37 to 2.84, *I^2^* = 90%, *p* = 0.01; last follow-up, *n* = 621, MD = 1.26, 95% CI: 0.31 to 2.21, *I^2^* = 88%, *p* = 0.009; very low certainty; Figure 6).

Additional stratified analyses revealed that the effect estimates for all subgroups remained congruent with the overall result. However, the subgroup of RCTs included only one eligible study, precluding a meaningful statistical analysis (Table 4; Supplementary Figures S5, S6).

### ODI scores

**Figure 7:**
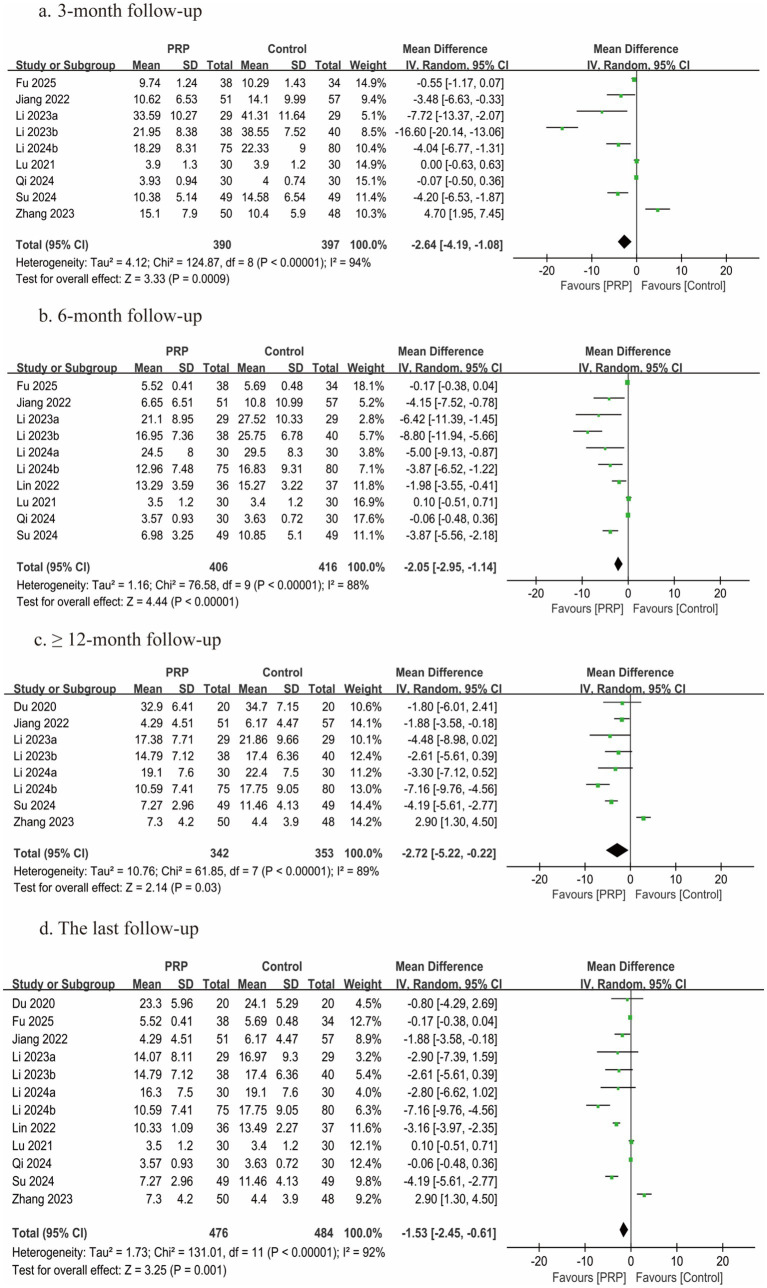
Forest plot of the meta-analysis comparing ODI scores. **(a)** The duration of follow-up was 3 months, **(b)** the duration of follow-up was 6 months, **(c)** the duration of follow-up was longer than 12 months, and **(d)** the last follow-up.

Twelve studies (2, 25–35) comprising 960 patients reported ODI scores; each study was classified as having a moderate risk of bias. Nine studies (2, 25, 27–31, 33, 34) reported ODI scores at 3 months, 10 studies (25–34) reported ODI scores at 6 months, and eight studies (2, 26, 27, 29–31, 33, 35) reported ODI scores at ≥12 months post-treatment. In all follow-up periods (3, 6, and ≥12 months) and at the final follow-up, statistically significant differences between the groups were found by the meta-analysis (3 months, *n* = 787, MD = −2.64, 95% CI: −4.19 to −1.08, *I^2^* = 94%, *p* = 0.0009; 6 months, *n* = 822, MD = −2.05, 95% CI: −2.95 to −1.14, *I^2^* = 88%, *p* < 0.00001; ≥12 months, *n* = 695, MD = −2.72, 95% CI: −5.22 to −0.22, *I^2^* = 89%, *p* = 0.03; last follow-up, *n* = 960, MD = −1.53, 95% CI: −2.45 to −0.61, *I^2^* = 92%, *p* = 0.001; very low certainty) (Figure 7).

Subgroup analyses were performed based on study design and patient age. The pooled results revealed no statistically significant impact on ODI scores in either the RCT subgroup (*n* = 198, MD = −1.73, 95% CI: −4.90 to 1.45, *I^2^* = 93%, *p* = 0.29) or the subgroup of patients aged < 40 years (*n* = 210, MD = −1.24, 95% CI: −3.18 to 0.71, *I^2^* = 54%, *p* = 0.21) (Table 4; Supplementary Figures S7, S8).

### Disk height

**Figure 8:**
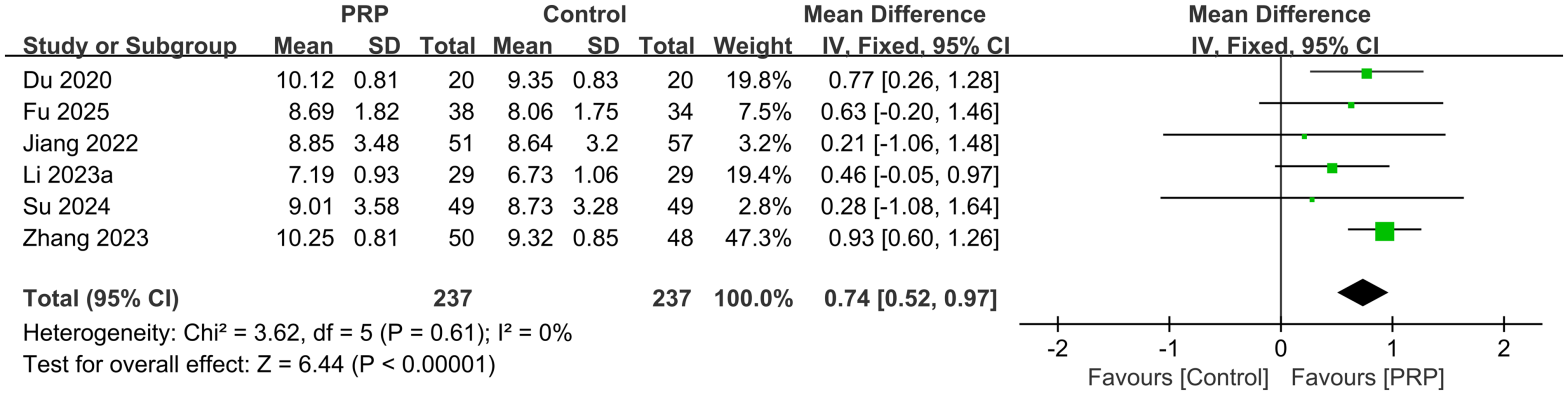
Forest plot of the meta-analysis comparing the disc height.

Six studies (2, 25, 29, 30, 33, 35) involving 474 participants assessed disk height outcomes; each study was classified as having a moderate risk of bias. Due to minimal heterogeneity (*p* = 0.61, *I^2^* = 0%), a fixed-effects model was used, demonstrating a statistically significant difference (*n* = 474, MD = 0.74, 95% Cl: 0.52 to 0.97, *I^2^* = 0%, *p* < 0.00001; low certainty; Figure 8).

### Complication

**Figure 9:**
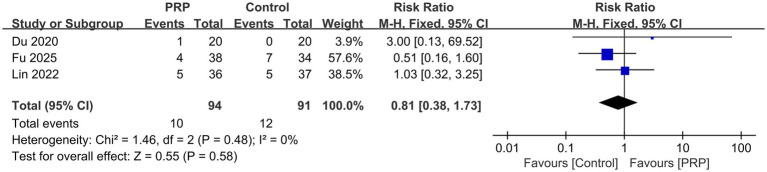
Forest plot of the meta-analysis comparing the complications.

Complications were reported by three studies (25, 32, 35), including 185 patients; each study was judged to have a moderate risk of bias. With low heterogeneity (*p* = 0.48, *I^2^* = 0%) and a fixed-effects model, the analysis showed no discernible variation in the incidence of complications between the groups (*n* = 185, RR = 0.81, 95% Cl: 0.38 to 1.73, *I^2^* = 0%, *p* = 0.58; very low certainty; Figure 9).

### Recurrence

**Figure 10:**
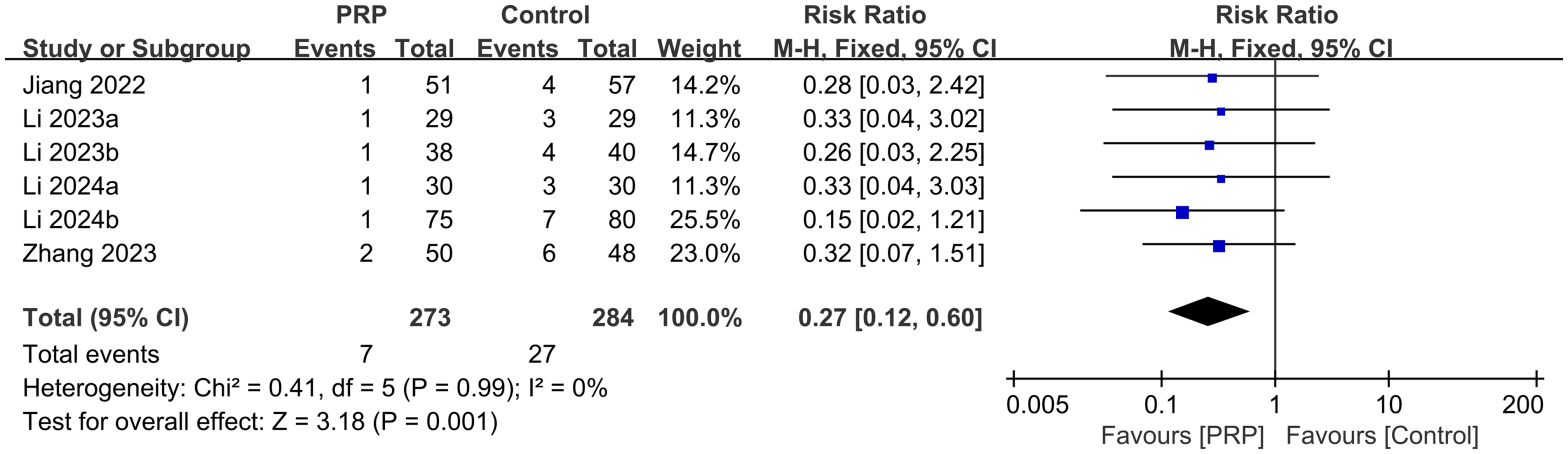
Forest plot of the meta-analysis comparing the recurrence.

Recurrence was reported by six studies (2, 26, 27, 30, 31, 33), including 557 patients; each study was judged to have a moderate risk of bias. With low heterogeneity (*p* = 0.99, *I^2^* = 0%) and a fixed-effects model, the analysis showed discernible variation in the incidence of recurrence between groups (*n* = 557, RR = 0.27, 95% Cl: 0.12 to 0.60, *I^2^* = 0%, *p* = 0.001; low certainty; Figure 10).

### Sensitivity analysis

The reliability of outcome measures, including back VAS scores, leg VAS scores, JOA scores, ODI scores, disk height, complications, and recurrence, was assessed via a sensitivity analysis. For back VAS scores, Su et al. (29) introduced notable heterogeneity, which was significantly reduced upon its exclusion (*p* = 0.11, *I^2^* = 36%). Similarly, removing Su et al. (29) resolved significant heterogeneity observed in leg VAS scores analyses (*p* = 0.34, *I^2^* = 12%). For JOA scores, Zhang et al. (2) introduced notable heterogeneity, which was significantly reduced upon its exclusion (*p* = 0.64, *I^2^* = 0%). Sensitivity analyses of other outcomes confirmed the stability of pooled effect estimates. Complete sensitivity analysis data are provided in Supplementary Tables S1–S7.

### Publication bias

Egger’s regression tests and funnel plots used to analyze publication bias revealed no discernible biases for the outcomes under consideration. Detailed results are provided in Supplementary Table S8 and Supplementary Figures S9, S10.

### GRADE evaluation

Based on the principles of the GRADE evaluation, we assessed the quality of the evidence in terms of back VAS scores, leg VAS scores, JOA scores, ODI scores, disk height, complications, and recurrence. As the majority of the included trials were non-randomized controlled trials and implemented minimal blinding or allocation concealment, this resulted in an initial low rating and further downgrading. As shown in Supplementary Table S9, the evidence for the disk height and recurrence was classified as low quality, while the other evidence was of very low quality.

## Discussion

### Summary of evidence

This meta-analysis systematically reviewed 12 eligible studies (2, 25–35) comprising 960 patients with LDH. Regarding back VAS scores, leg VAS scores, JOA scores, ODI scores, disk height, and recurrence, the combined results showed that patients undergoing endoscopic surgery combined with PRP therapy had noticeably better results than controls. The robustness of these findings was verified through sensitivity analyses.

### Interpretation of findings

Lower back pain and radiating leg pain are major clinical symptoms in LDH patients. Evaluating pain changes is therefore essential. VAS scores are widely used in clinical studies due to their simplicity and patient acceptance. Jiang et al. (33) reported that PRP therapy combined with endoscopic surgery significantly reduced back and leg VAS scores. Similarly, Xu et al. (36) observed effective pain relief following transforaminal PRP injections. Hirase et al. (37) conducted a meta-analysis of five retrospective studies and concluded that intradiscal PRP injection for degenerative lumbar disk degeneration resulted in a statistically significant improvement in VAS scores. This meta-analysis reinforces our observation that PRP is a safe adjunctive approach. Lumbar intervertebral disks consist of cartilaginous endplates, nucleus pulposus, and annulus fibrosus and bear significant stress during spinal movements. LDH occurs when the annulus fibrosus partially or fully ruptures, allowing nucleus pulposus protrusion. This protrusion compresses adjacent nerve roots, causing impaired blood supply, nerve root edema, altered ion-channel expression, and heightened pain sensitivity. Furthermore, ruptured nucleus pulposus material disrupts the blood–nucleus barrier, triggering cellular and humoral immune responses upon blood contact (38–40). The mechanical compression of nerve roots caused by the herniated nucleus pulposus initiates inflammatory responses, which are crucial in the development of pain. Recent findings indicate that PRP plays a significant role in alleviating inflammation through the downregulation of pro-inflammatory cytokines (41, 42). Kim et al. (43) showed that PRP enhances the expression of essential extracellular matrix genes, such as collagen type II and aggrecan, which are inhibited by inflammatory mediators, while reducing the expression of degradative enzymes such as COX-2 and MMP-3. Consequently, PRP therapy could alleviate inflammatory damage within the nucleus pulposus, thereby enhancing the overall microenvironment of intervertebral disks. Additional studies suggest that PRP contains abundant growth factors promoting nerve regeneration, offering analgesic and anti-inflammatory effects (44).

The LDH patients are assessed using the JOA score system, which includes clinical indicators, subjective symptoms, daily living activities, and bladder function. An RCT involving 40 LDH patients undergoing percutaneous endoscopic lumbar discectomy, with or without adjunctive PRP injection, reported significantly elevated JOA scores at 24-month follow-up in patients treated with the PRP group (35). Du et al. proposed that surgical intervention primarily restores disk mechanics, whereas supplementary PRP treatment enhances disk biological function (35). Similarly, Li et al. (26) observed improvements in JOA scores across both groups, with notably greater gains among patients receiving PRP therapy, suggesting that combined PRP treatment might enhance lumbar function and physical performance.

The ODI score is a commonly used clinical tool for assessing how patients’ lower back pain affects functional activities. One prospective randomized controlled study reported significant ODI improvements over a 1-year follow-up among LDH patients receiving transforaminal injections of either PRP or steroids. Although intergroup differences were non-significant, the comparable effectiveness and safety profile indicated that PRP injection might be a preferable treatment option (36). Su et al. (29) concluded that injecting PRP into the intervertebral disk and around the nerve roots can rapidly exert anti-inflammatory effects and promote tissue repair, thereby enhancing patients’ quality of life and work.

The disk height is quantified radiologically through standardized imaging protocols as an objective measure of lumbar intervertebral space. Current evidence suggests that minimally invasive surgery combined with PRP therapy demonstrates superior disk height recovery compared to surgery alone. The LDH typically occurs after annulus fibrosus rupture due to disk degeneration (45). As avascular tissues, intervertebral disks depend primarily on nutrient diffusion through endplates and the annulus fibrosus, limiting their self-repair capacity post-degeneration. Therefore, enhancing intervertebral disk repair has become an important research topic. PRP is biologically safe, easy to prepare, and promotes disk cell proliferation and extracellular matrix synthesis (46–48). Li et al. (30) reported that minimally invasive surgery combined with PRP achieved maximal restoration of disk height. Although minimally invasive surgery effectively removes disk tissue, it may compromise disk integrity, leading to reduced disk height, secondary instability, and potentially accelerated disk degeneration. PRP therapy applied during surgery releases numerous growth factors, which promote tissue regeneration, improve microcirculation, and facilitate disk repair. Optimal clinical outcomes require balancing surgical decompression with maximal preservation of normal disk tissue, creating an ideal biological environment for PRP repair. This combined treatment provides dual benefits: precise surgical removal of pathological lesions, biomechanical restoration, PRP-enhanced disk regeneration, and biological repair.

In our comprehensive analysis, the rates of complication exhibited no statistically significant differences among the groups examined. Several negative occurrences, including dural tears and nerve root contusions, were recorded in patients undergoing PRP treatment, potentially linked to the procedural learning curve associated with the administration of PRP. One of the key findings of our investigation is the reduced recurrence in patients undergoing endoscopic surgery combined with PRP treatment. Primary surgery may alter local anatomy, induce epidural fibrosis, and promote scar tissue formation, all of which increase the technical difficulty and risks of revision surgery. Jiang et al. (33) reported only one recurrence requiring reoperation among 51 cases over a 1-year follow-up. Similarly, Zhang et al. (2) demonstrated that PRP administration following endoscopic surgery significantly lowers the risk of recurrence and the need for reoperation.

## Strengths and limitations

Our study differs from other meta-analyses in three main aspects (49–51). First, we included all studies evaluating endoscopic surgery combined with PRP therapy for LDH from eight databases. Expanding the search across multiple databases enhances the comprehensiveness of the evidence, reduces selection bias, and strengthens the robustness of the meta-analysis conclusions. Second, unlike previous studies that only analyzed the clinical application effects of PRP therapy based on patients’ subjective feelings, which lack scientific validity and reliability, our study comprehensively evaluates the efficacy of PRP by considering VAS scores, JOA scores, ODI scores, disk height, complications, and recurrence. Finally, we performed a GRADE assessment of the evidence level for each outcome measure, providing a reference for clinical practice.

This research has several limitations that should be considered when interpreting the results. The preliminary evaluation shows that the studies included in the study had inferior methodological quality, which could affect the reliability of the findings. Second, while all investigations used PRP therapy, variations in preparation methodologies, effective concentrations, dosages, and administration techniques may influence the results. Third, specific outcome measures depended on subjective scales, which may have been affected by differing patient and researcher interpretations. Fourth, the studies reviewed exhibited a deficiency in long-term follow-up, which restricts a thorough assessment of the clinical efficacy of PRP for LDH. Future studies should incorporate objective assessments of disk remodeling or degeneration alongside patient-reported outcomes, including quality-of-life metrics and satisfaction. Ultimately, the majority of studies have been carried out in China, which may restrict the wider clinical implementation of PRP. To improve the credibility of the results, large-scale, multicenter RCTs in different regions should be conducted in the future.

## Conclusion

Current evidence suggests that endoscopic surgery combined with PRP is a promising treatment for LDH. However, the existing studies have methodological limitations that warrant cautious interpretation. Future research should employ well-designed, multicenter RCTs to rigorously evaluate its efficacy and safety for LDH.

## Data Availability

The datasets presented in this study can be found in online repositories. The names of the repository/repositories and accession number(s) can be found in the article/Supplementary material.
