# Supplementary material for: The efficacy and safety of endoscopic surgery combined with platelet-rich plasma for lumbar disk herniation: a systematic review and meta-analysis
Source: Front Med (Lausanne). 2025 Dec 5;12:1697117. doi: 10.3389/fmed.2025.1697117 (PMC12714916; doi:10.3389/fmed.2025.1697117)
Supplement: Supplementary file 1 [file Supplementary_file_1.pdf]

Supplementary Material

The efficacy and safety of endoscopic surgery combined with platelet-rich plasma for lumbar disc herniation: a systematic review and meta-analysis

Search strategies 2
Fig. S1. Subgroup analysis of back VAS scores by study design. 4
Fig. S2. Subgroup analysis of back VAS scores by patient age. 5
Fig. S3. Subgroup analysis of leg VAS scores by study design. 6
Fig. S4. Subgroup analysis of leg VAS scores by patient age. 7
Fig. S5. Subgroup analysis of JOA scores by study design. 8
Fig. S6. Subgroup analysis of JOA scores by patient age. 9
Fig. S7. Subgroup analysis of ODI scores by study design. 10
Fig. S8. Subgroup analysis of ODI scores by patient age. 11
Table S1. Sensitivity analysis for back VAS scores. 12
Table S2. Sensitivity analysis for leg VAS scores. 13
Table S3. Sensitivity analysis for JOA scores. 14
Table S4. Sensitivity analysis for ODI scores. 15
Table S5. Sensitivity analysis for disc height. 16
Table S6. Sensitivity analysis for complication. 17
Table S7. Sensitivity analysis for recurrence. 18
Fig. S9. Forest plot of back VAS scores. 19
Fig. S10. Forest plot of ODI scores. 20
Table S8. Publication bias of the included studies. 21
Table S9. GRADE evaluation of evidence quality. 22

Search strategies

PubMed:

#1: "lumbar disc herniation"[Title/Abstract] OR "lumbar disc protrusion"[Title/Abstract] OR "intervertebral disc displacement"[Title/Abstract] OR "lumber disc herniation"[Title/Abstract] OR "LDH"[Title/Abstract]

#2: "platelet-rich plasma"[Title/Abstract] OR "platelet-rich"[Title/Abstract] OR "platelet rich plasma"[Title/Abstract]

#3: #1 AND #2

Web of Science:

#1: lumbar disc herniation (Topic) OR lumbar disc protrusion (Topic) OR intervertebral disc displacement (Topic) OR lumber disc herniation (Topic) OR LDH (Topic)

#2: platelet-rich plasma (Topic) OR platelet-rich (Topic) OR platelet rich plasma (Topic)

#3: #1 AND #2

EMBASE:

#1: 'lumbar disc herniation':ti,ab,kw OR 'lumbar disc protrusion':ti,ab,kw OR 'intervertebral disc displacement':ti,ab,kw OR 'lumber disc herniation':ti,ab,kw OR 'LDH':ti,ab,kw

#2: 'platelet-rich plasma':ti,ab,kw OR 'platelet-rich':ti,ab,kw OR 'platelet rich plasma':ti,ab,kw

#3: #1 AND #2

Cochrane Library：

#1: 'lumbar disc herniation':ti,ab,kw OR 'lumbar disc protrusion':ti,ab,kw OR 'intervertebral disc displacement':ti,ab,kw OR 'lumber disc herniation':ti,ab,kw OR 'LDH':ti,ab,kw

#2: 'platelet-rich plasma':ti,ab,kw OR 'platelet-rich':ti,ab,kw OR 'platelet rich plasma':ti,ab,kw

#3: #1 AND #2

Chinese National Knowledge Infrastructure：

(SU='腰椎间盘突出症' + '腰椎间盘突出' + '腰痛' + '腰痹') AND (SU='富血小板血浆' + '富血小板')

Chinese Science and Technology Journal database：

M=(腰椎间盘突出症 OR 腰椎间盘突出 OR 腰痛 OR 腰痹) AND M=(富血小板血浆 OR 富血小板)

WanFang database：

主题:(“腰椎间盘突出症” or “腰椎间盘突出” or “腰痛” or “腰痹”) and 主题:(“富血小板血浆” or “富血小板”)

Chinese Biological Literature database：

("腰椎间盘突出症"[摘要:智能] OR "腰椎间盘突出"[摘要:智能] OR "腰痛"[摘要:智能] OR "腰痹"[摘要:智能]) AND ("富血小板血浆"[摘要:智能] OR "富血小板"[摘要:智能])

Study or Subgroup	PRP Mean	PRP SD	PRP Total	Control Mean	Control SD	Control Total	Weight	Mean Difference IV, Random, 95% CI
**1.1.1 RCT**								
Du 2020	2.35	0.93	20	2.6	0.94	20	3.7%	-0.25 [-0.83, 0.33]
Qi 2024	0.4	0.56	30	0.37	0.62	30	8.4%	0.03 [-0.27, 0.33]
Su 2024	1.19	0.49	49	1.84	0.79	49	9.4%	-0.65 [-0.91, -0.39]
**Subtotal (95% CI)**			**99**			**99**	**21.5%**	**-0.30 [-0.78, 0.19]**
Heterogeneity: Tau² = 0.15; Chi² = 11.42, df = 2 (P = 0.003); I² = 82%								
Test for overall effect: Z = 1.20 (P = 0.23)								
**1.1.2 Non-RCT**								
Fu 2025	1.98	0.26	38	2.04	0.13	34	14.0%	-0.06 [-0.15, 0.03]
Jiang 2022	0.82	0.56	51	1.11	0.82	57	9.3%	-0.29 [-0.55, -0.03]
Li 2023a	0.61	0.48	29	0.82	0.58	29	9.0%	-0.21 [-0.48, 0.06]
Li 2023b	1.14	0.81	38	1.34	0.84	40	6.8%	-0.20 [-0.57, 0.17]
Li 2024a	1.4	0.8	30	1.6	0.8	30	6.1%	-0.20 [-0.60, 0.20]
Li 2024b	0.55	0.7	75	0.83	0.84	80	9.9%	-0.28 [-0.52, -0.04]
Lin 2022	0.86	0.56	36	0.91	0.37	37	10.6%	-0.05 [-0.27, 0.17]
Lu 2021	0.5	0.53	30	0.6	0.62	30	8.5%	-0.10 [-0.39, 0.19]
Zhang 2023	0.9	0.8	50	1.8	1.7	48	4.3%	-0.90 [-1.43, -0.37]
**Subtotal (95% CI)**			**377**			**385**	**78.5%**	**-0.18 [-0.30, -0.07]**
Heterogeneity: Tau² = 0.01; Chi² = 14.30, df = 8 (P = 0.07); I² = 44%								
Test for overall effect: Z = 3.18 (P = 0.001)								
**Total (95% CI)**			**476**			**484**	**100.0%**	**-0.23 [-0.36, -0.10]**
Heterogeneity: Tau² = 0.03; Chi² = 30.35, df = 11 (P = 0.001); I² = 64%								
Test for overall effect: Z = 3.46 (P = 0.0005)								
Test for subgroup differences: Chi² = 0.20, df = 1 (P = 0.65), I² = 0%								

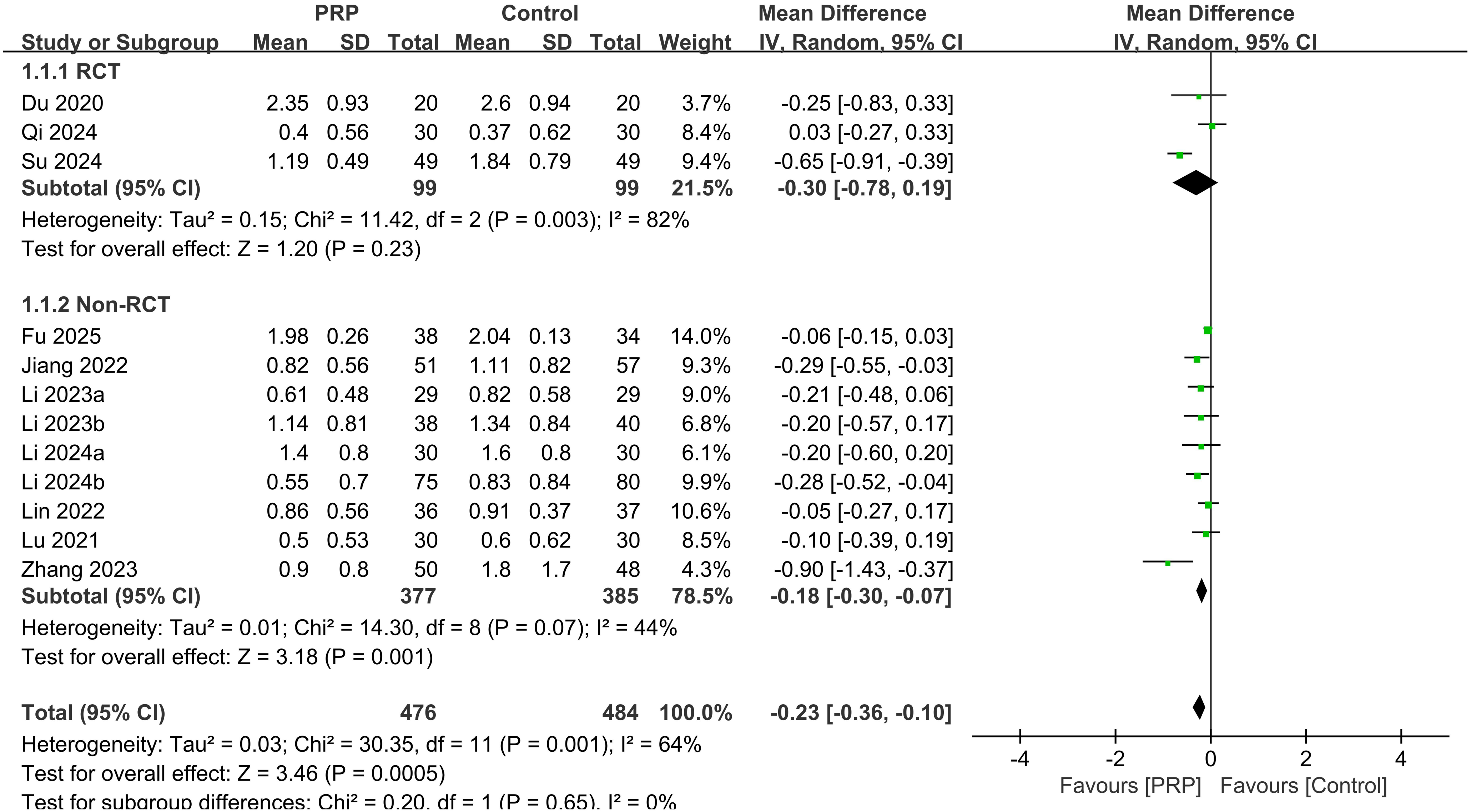

Fig. S1. Subgroup analysis of back VAS scores by study design.

Study or Subgroup	PRP Mean	PRP SD	PRP Total	Control Mean	Control SD	Control Total	Weight	Mean Difference IV, Random, 95% CI
**1.2.1 <40 years old**								
Fu 2025	1.98	0.26	38	2.04	0.13	34	14.7%	-0.06 [-0.15, 0.03]
Li 2023b	1.14	0.81	38	1.34	0.84	40	7.6%	-0.20 [-0.57, 0.17]
Li 2024a	1.4	0.8	30	1.6	0.8	30	6.8%	-0.20 [-0.60, 0.20]
**Subtotal (95% CI)**			**106**			**104**	**29.2%**	**-0.07 [-0.16, 0.01]**
Heterogeneity: Tau² = 0.00; Chi² = 0.91, df = 2 (P = 0.63); I² = 0%								
Test for overall effect: Z = 1.66 (P = 0.10)								
**1.2.2 ⩾40 years old**								
Du 2020	2.35	0.93	20	2.6	0.94	20	4.3%	-0.25 [-0.83, 0.33]
Jiang 2022	0.82	0.56	51	1.11	0.82	57	10.2%	-0.29 [-0.55, -0.03]
Li 2023a	0.61	0.48	29	0.82	0.58	29	9.9%	-0.21 [-0.48, 0.06]
Li 2024b	0.55	0.7	75	0.83	0.84	80	10.7%	-0.28 [-0.52, -0.04]
Lin 2022	0.86	0.56	36	0.91	0.37	37	11.4%	-0.05 [-0.27, 0.17]
Qi 2024	0.4	0.56	30	0.37	0.62	30	9.2%	0.03 [-0.27, 0.33]
Su 2024	1.19	0.49	49	1.84	0.79	49	10.2%	-0.65 [-0.91, -0.39]
Zhang 2023	0.9	0.8	50	1.8	1.7	48	4.9%	-0.90 [-1.43, -0.37]
**Subtotal (95% CI)**			**340**			**350**	**70.8%**	**-0.29 [-0.48, -0.11]**
Heterogeneity: Tau² = 0.05; Chi² = 21.60, df = 7 (P = 0.003); I² = 68%								
Test for overall effect: Z = 3.09 (P = 0.002)								
**Total (95% CI)**			**446**			**454**	**100.0%**	**-0.24 [-0.38, -0.10]**
Heterogeneity: Tau² = 0.03; Chi² = 30.21, df = 10 (P = 0.0008); I² = 67%								
Test for overall effect: Z = 3.38 (P = 0.0007)								
Test for subgroup differences: Chi² = 4.32, df = 1 (P = 0.04), I² = 76.8%								

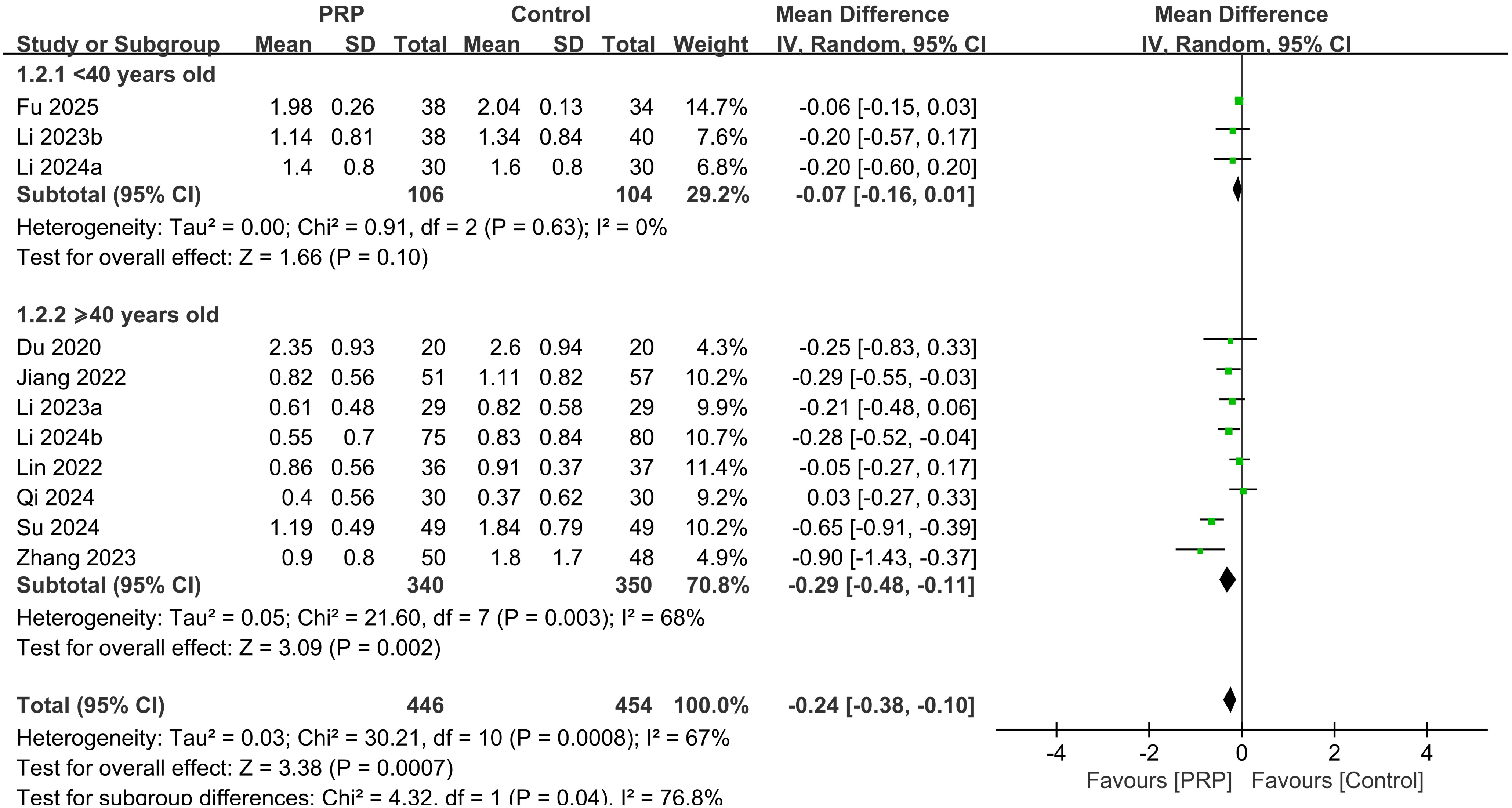

Fig. S2. Subgroup analysis of back VAS scores by patient age.

Study or Subgroup	PRP Mean	PRP SD	PRP Total	Control Mean	Control SD	Control Total	Weight	Mean Difference IV, Random, 95% CI
**2.1.1 RCT**								
Su 2024	1.2	0.65	49	2.01	0.73	49	18.8%	-0.81 [-1.08, -0.54]
**Subtotal (95% CI)**			**49**			**49**	**18.8%**	**-0.81 [-1.08, -0.54]**
Heterogeneity: Not applicable								
Test for overall effect: Z = 5.80 (P < 0.00001)								
**2.1.2 Non-RCT**								
Jiang 2022	0.75	0.74	51	1.09	0.93	57	17.5%	-0.34 [-0.66, -0.02]
Li 2023a	1.29	0.64	29	1.63	0.84	29	15.4%	-0.34 [-0.72, 0.04]
Li 2024a	2	0.9	30	2.2	0.8	30	14.1%	-0.20 [-0.63, 0.23]
Li 2024b	0.97	0.79	75	1.34	1.02	80	18.4%	-0.37 [-0.66, -0.08]
Zhang 2023	1.1	0.9	50	1	1	48	15.6%	0.10 [-0.28, 0.48]
**Subtotal (95% CI)**			**235**			**244**	**81.2%**	**-0.25 [-0.42, -0.09]**
Heterogeneity: Tau² = 0.00; Chi² = 4.55, df = 4 (P = 0.34); I² = 12%								
Test for overall effect: Z = 2.97 (P = 0.003)								
**Total (95% CI)**			**284**			**293**	**100.0%**	**-0.35 [-0.60, -0.10]**
Heterogeneity: Tau² = 0.07; Chi² = 16.46, df = 5 (P = 0.006); I² = 70%								
Test for overall effect: Z = 2.71 (P = 0.007)								
Test for subgroup differences: Chi² = 11.63, df = 1 (P = 0.0006), I² = 91.4%								

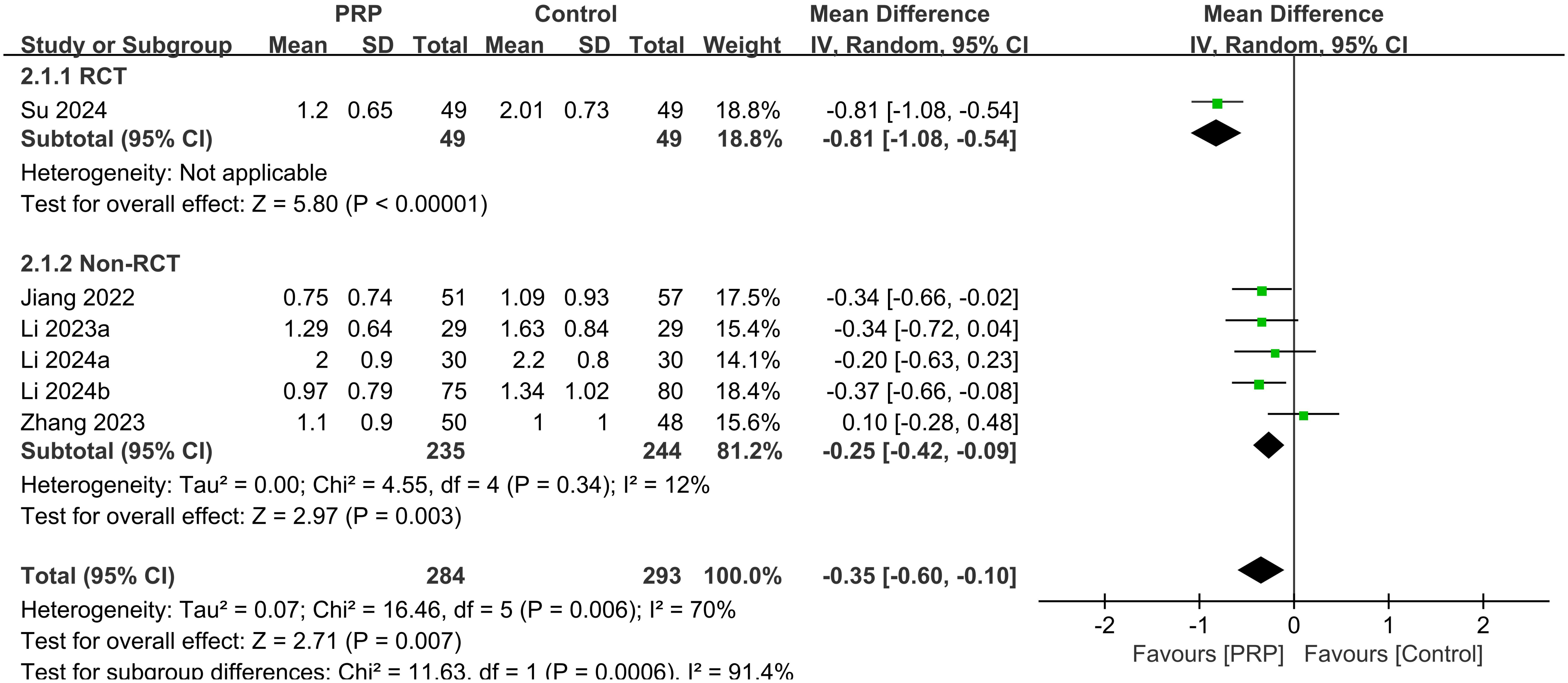

Fig. S3. Subgroup analysis of leg VAS scores by study design.

Study or Subgroup	PRP Mean	PRP SD	PRP Total	Control Mean	Control SD	Control Total	Weight	Mean Difference IV, Random, 95% CI
**2.2.1 <40 years old**								
Li 2024a	2	0.9	30	2.2	0.8	30	14.1%	-0.20 [-0.63, 0.23]
**Subtotal (95% CI)**			**30**			**30**	**14.1%**	**-0.20 [-0.63, 0.23]**
Heterogeneity: Not applicable								
Test for overall effect: Z = 0.91 (P = 0.36)								
**2.2.2 ⩾40 years old**								
Jiang 2022	0.75	0.74	51	1.09	0.93	57	17.5%	-0.34 [-0.66, -0.02]
Li 2023a	1.29	0.64	29	1.63	0.84	29	15.4%	-0.34 [-0.72, 0.04]
Li 2024b	0.97	0.79	75	1.34	1.02	80	18.4%	-0.37 [-0.66, -0.08]
Su 2024	1.2	0.65	49	2.01	0.73	49	18.8%	-0.81 [-1.08, -0.54]
Zhang 2023	1.1	0.9	50	1	1	48	15.6%	0.10 [-0.28, 0.48]
**Subtotal (95% CI)**			**254**			**263**	**85.9%**	**-0.37 [-0.65, -0.08]**
Heterogeneity: Tau² = 0.08; Chi² = 15.62, df = 4 (P = 0.004); I² = 74%								
Test for overall effect: Z = 2.53 (P = 0.01)								
**Total (95% CI)**			**284**			**293**	**100.0%**	**-0.35 [-0.60, -0.10]**
Heterogeneity: Tau² = 0.07; Chi² = 16.46, df = 5 (P = 0.006); I² = 70%								
Test for overall effect: Z = 2.71 (P = 0.007)								
Test for subgroup differences: Chi² = 0.40, df = 1 (P = 0.53), I² = 0%								

Mean Difference
IV, Random, 95% CI

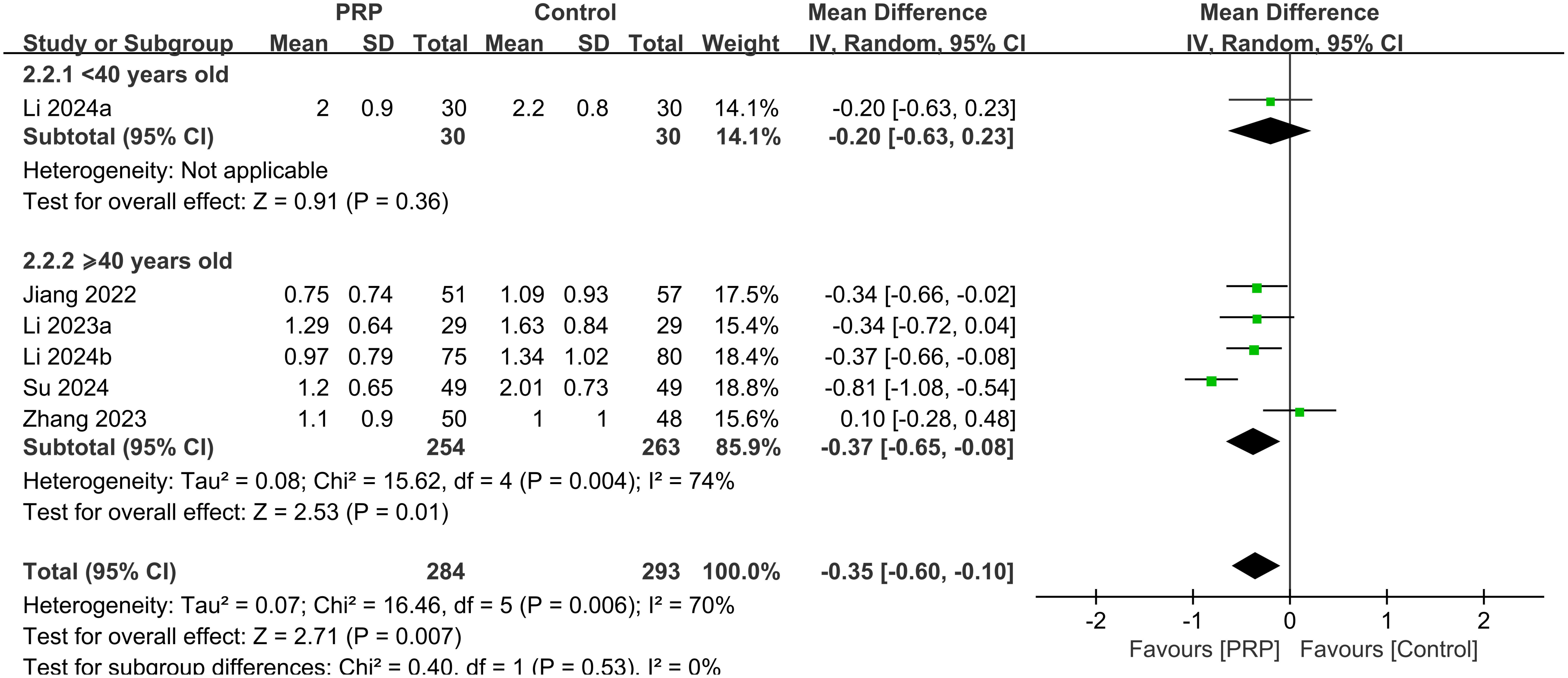

Fig. S4. Subgroup analysis of leg VAS scores by patient age.

Study or Subgroup	PRP Mean	PRP SD	PRP Total	Control Mean	Control SD	Control Total	Weight	Mean Difference IV, Random, 95% CI
**3.1.1 RCT**								
Du 2020	23.65	2.54	20	22.45	2.06	20	10.9%	1.20 [-0.23, 2.63]
**Subtotal (95% CI)**			**20**			**20**	**10.9%**	**1.20 [-0.23, 2.63]**
Heterogeneity: Not applicable								
Test for overall effect: Z = 1.64 (P = 0.10)								
**3.1.2 Non-RCT**								
Fu 2025	23.15	1.63	38	22.69	1.84	34	13.1%	0.46 [-0.35, 1.27]
Li 2023a	23.34	1.84	29	22.59	1.97	29	12.5%	0.75 [-0.23, 1.73]
Li 2023b	23.74	1.84	38	23.03	1.69	40	13.1%	0.71 [-0.08, 1.50]
Li 2024a	25.5	2.2	30	24.7	2.1	30	12.1%	0.80 [-0.29, 1.89]
Li 2024b	25.33	2.35	75	24.28	2.48	80	13.2%	1.05 [0.29, 1.81]
Lu 2021	23.9	1.3	30	23.8	1.5	30	13.4%	0.10 [-0.61, 0.81]
Zhang 2023	26.6	3	50	21.2	3.1	48	11.7%	5.40 [4.19, 6.61]
**Subtotal (95% CI)**			**290**			**291**	**89.1%**	**1.27 [0.23, 2.31]**
Heterogeneity: $Tau^2 = 1.76$; $Chi^2 = 59.66$, df = 6 ($P < 0.00001$); $I^2 = 90\%$								
Test for overall effect: Z = 2.39 (P = 0.02)								
**Total (95% CI)**			**310**			**311**	**100.0%**	**1.26 [0.31, 2.21]**
Heterogeneity: $Tau^2 = 1.62$; $Chi^2 = 59.76$, df = 7 ($P < 0.00001$); $I^2 = 88\%$								
Test for overall effect: Z = 2.60 (P = 0.009)								
Test for subgroup differences: $Chi^2 = 0.01$, df = 1 (P = 0.94), $I^2 = 0\%$								

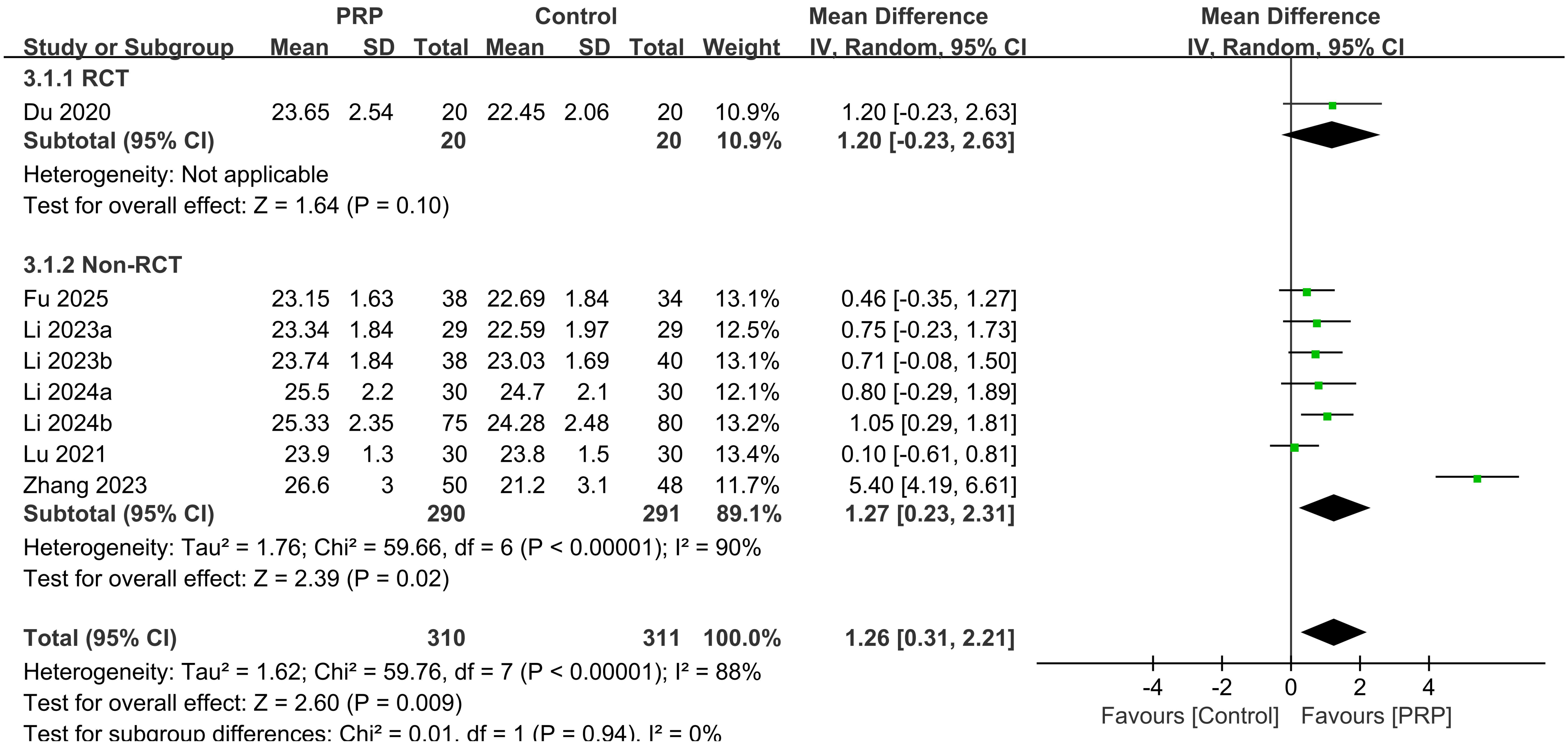

Fig. S5. Subgroup analysis of JOA scores by study design.

Study or Subgroup	PRP Mean	PRP SD	PRP Total	Control Mean	Control SD	Control Total	Weight	Mean Difference IV, Random, 95% CI
**3.2.1 <40 years old**								
Fu 2025	23.15	1.63	38	22.69	1.84	34	15.0%	0.46 [-0.35, 1.27]
Li 2023b	23.74	1.84	38	23.03	1.69	40	15.1%	0.71 [-0.08, 1.50]
Li 2024a	25.5	2.2	30	24.7	2.1	30	14.0%	0.80 [-0.29, 1.89]
**Subtotal (95% CI)**			**106**			**104**	**44.1%**	**0.63 [0.13, 1.13]**
Heterogeneity: $Tau^2 = 0.00$; $Chi^2 = 0.30$, df = 2 (P = 0.86); $I^2 = 0\%$								
Test for overall effect: Z = 2.48 (P = 0.01)								
**3.2.2 ⩾40 years old**								
Du 2020	23.65	2.54	20	22.45	2.06	20	12.7%	1.20 [-0.23, 2.63]
Li 2023a	23.34	1.84	29	22.59	1.97	29	14.4%	0.75 [-0.23, 1.73]
Li 2024b	25.33	2.35	75	24.28	2.48	80	15.2%	1.05 [0.29, 1.81]
Zhang 2023	26.6	3	50	21.2	3.1	48	13.6%	5.40 [4.19, 6.61]
**Subtotal (95% CI)**			**174**			**177**	**55.9%**	**2.08 [0.09, 4.07]**
Heterogeneity: $Tau^2 = 3.80$; $Chi^2 = 42.85$, df = 3 (P < 0.00001); $I^2 = 93\%$								
Test for overall effect: Z = 2.05 (P = 0.04)								
**Total (95% CI)**			**280**			**281**	**100.0%**	**1.44 [0.37, 2.52]**
Heterogeneity: $Tau^2 = 1.83$; $Chi^2 = 52.59$, df = 6 (P < 0.00001); $I^2 = 89\%$								
Test for overall effect: Z = 2.63 (P = 0.009)								
Test for subgroup differences: $Chi^2 = 1.91$, df = 1 (P = 0.17), $I^2 = 47.8\%$								

Mean Difference
IV, Random, 95% CI

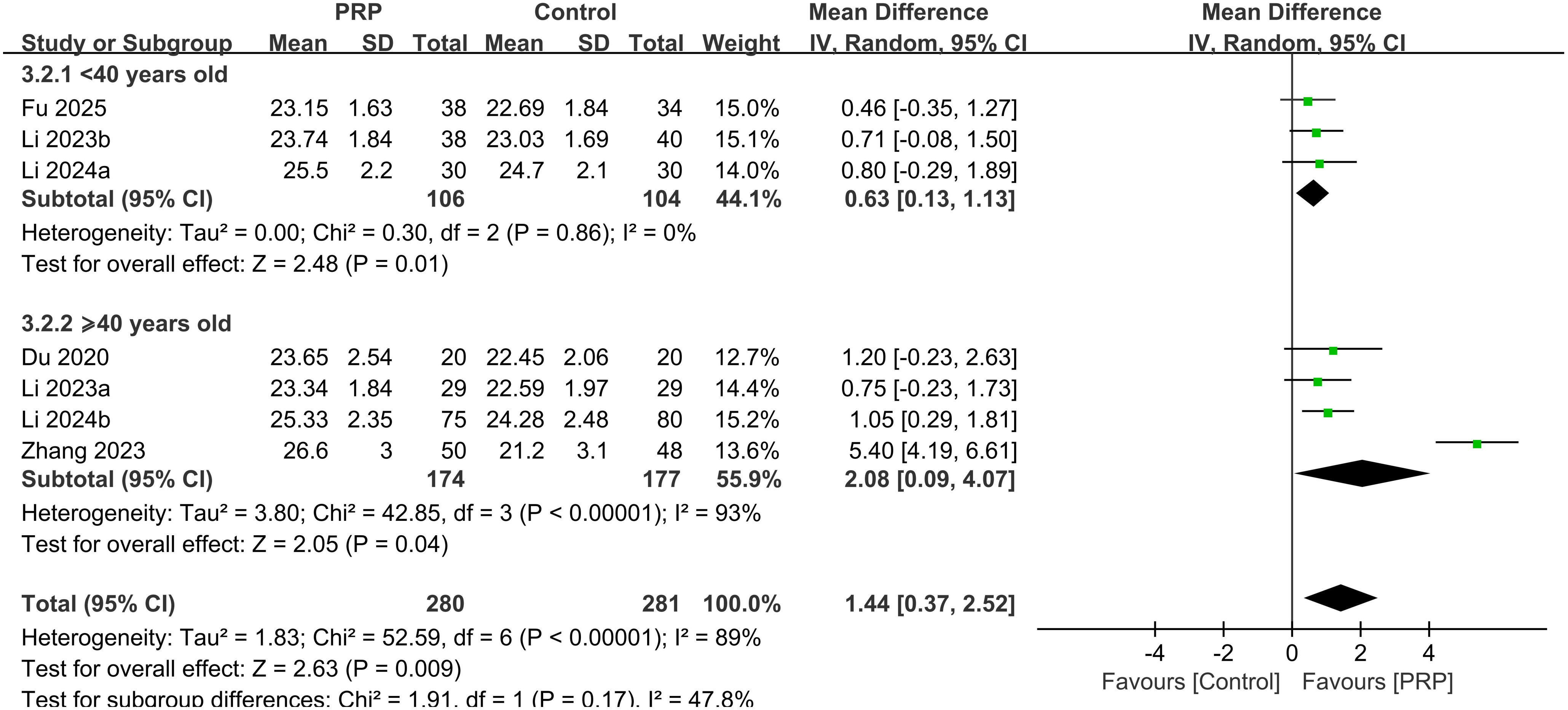

Fig. S6. Subgroup analysis of JOA scores by patient age.

Study or Subgroup	PRP Mean	PRP SD	PRP Total	Control Mean	Control SD	Control Total	Weight	Mean Difference IV, Random, 95% CI
**4.1.1 RCT**								
Du 2020	23.3	5.96	20	24.1	5.29	20	4.5%	-0.80 [-4.29, 2.69]
Qi 2024	3.57	0.93	30	3.63	0.72	30	12.4%	-0.06 [-0.48, 0.36]
Su 2024	7.27	2.96	49	11.46	4.13	49	9.8%	-4.19 [-5.61, -2.77]
**Subtotal (95% CI)**			**99**			**99**	**26.7%**	**-1.73 [-4.90, 1.45]**
Heterogeneity: Tau² = 6.84; Chi² = 29.82, df = 2 (P < 0.00001); I² = 93%								
Test for overall effect: Z = 1.07 (P = 0.29)								
**4.1.2 Non-RCT**								
Fu 2025	5.52	0.41	38	5.69	0.48	34	12.7%	-0.17 [-0.38, 0.04]
Jiang 2022	4.29	4.51	51	6.17	4.47	57	8.9%	-1.88 [-3.58, -0.18]
Li 2023a	14.07	8.11	29	16.97	9.3	29	3.2%	-2.90 [-7.39, 1.59]
Li 2023b	14.79	7.12	38	17.4	6.36	40	5.4%	-2.61 [-5.61, 0.39]
Li 2024a	16.3	7.5	30	19.1	7.6	30	4.0%	-2.80 [-6.62, 1.02]
Li 2024b	10.59	7.41	75	17.75	9.05	80	6.3%	-7.16 [-9.76, -4.56]
Lin 2022	10.33	1.09	36	13.49	2.27	37	11.6%	-3.16 [-3.97, -2.35]
Lu 2021	3.5	1.2	30	3.4	1.2	30	12.1%	0.10 [-0.51, 0.71]
Zhang 2023	7.3	4.2	50	4.4	3.9	48	9.2%	2.90 [1.30, 4.50]
**Subtotal (95% CI)**			**377**			**385**	**73.3%**	**-1.57 [-2.83, -0.31]**
Heterogeneity: Tau² = 2.60; Chi² = 101.13, df = 8 (P < 0.00001); I² = 92%								
Test for overall effect: Z = 2.45 (P = 0.01)								
**Total (95% CI)**			**476**			**484**	**100.0%**	**-1.53 [-2.45, -0.61]**
Heterogeneity: Tau² = 1.73; Chi² = 131.01, df = 11 (P < 0.00001); I² = 92%								
Test for overall effect: Z = 3.25 (P = 0.001)								
Test for subgroup differences: Chi² = 0.01, df = 1 (P = 0.93), I² = 0%								

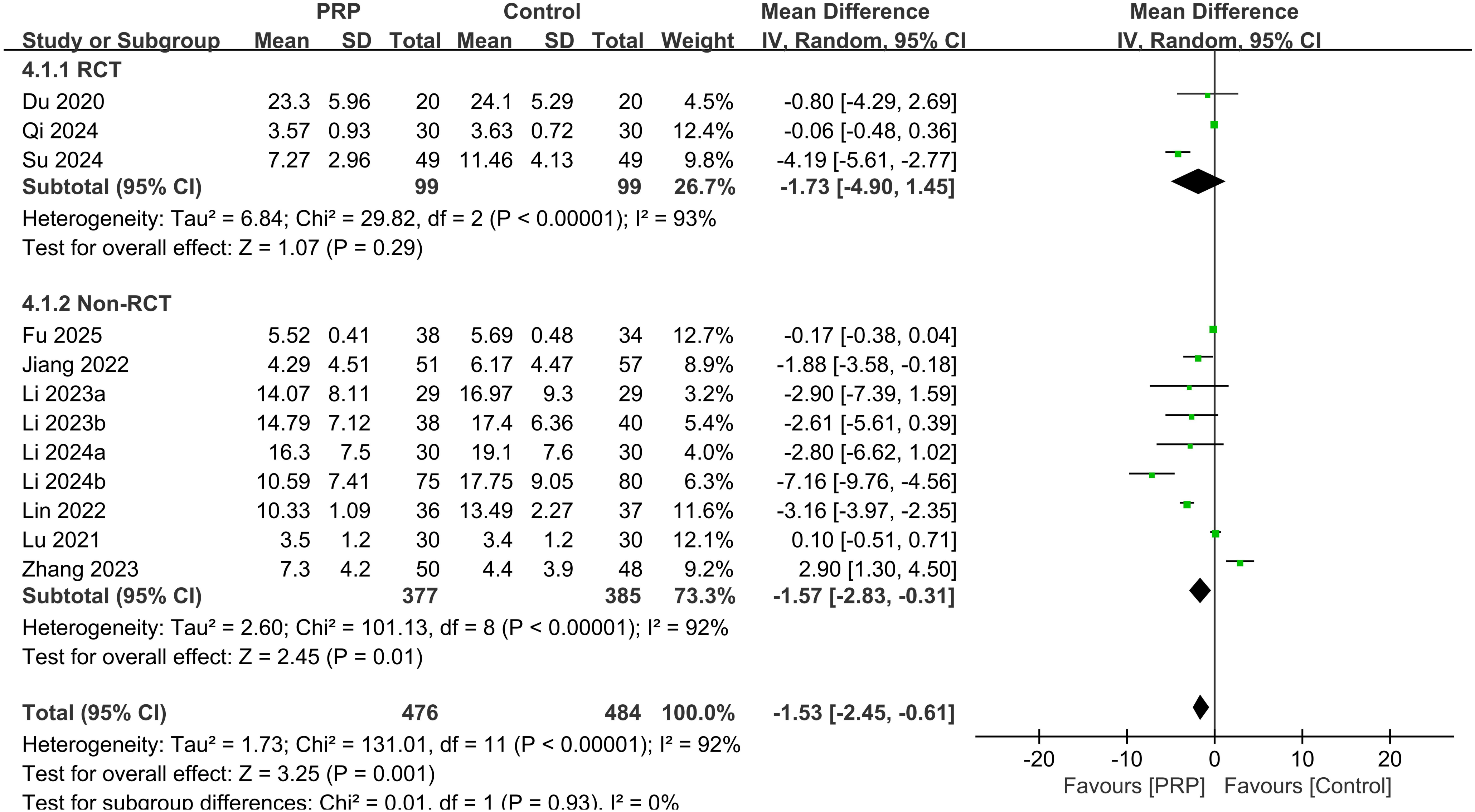

Fig. S7. Subgroup analysis of ODI scores by study design.

Study or Subgroup	PRP Mean	PRP SD	PRP Total	Control Mean	Control SD	Control Total	Weight	Mean Difference IV, Random, 95% CI
**4.2.1 <40 years old**								
Fu 2025	5.52	0.41	38	5.69	0.48	34	13.6%	-0.17 [-0.38, 0.04]
Li 2023b	14.79	7.12	38	17.4	6.36	40	6.6%	-2.61 [-5.61, 0.39]
Li 2024a	16.3	7.5	30	19.1	7.6	30	5.0%	-2.80 [-6.62, 1.02]
**Subtotal (95% CI)**			**106**			**104**	**25.2%**	**-1.24 [-3.18, 0.71]**
Heterogeneity: $Tau^2 = 1.70$; $Chi^2 = 4.33$, df = 2 (P = 0.12); $I^2 = 54\%$								
Test for overall effect: Z = 1.25 (P = 0.21)								
**4.2.2 ⩾40 years old**								
Du 2020	23.3	5.96	20	24.1	5.29	20	5.6%	-0.80 [-4.29, 2.69]
Jiang 2022	4.29	4.51	51	6.17	4.47	57	10.2%	-1.88 [-3.58, -0.18]
Li 2023a	14.07	8.11	29	16.97	9.3	29	4.0%	-2.90 [-7.39, 1.59]
Li 2024b	10.59	7.41	75	17.75	9.05	80	7.6%	-7.16 [-9.76, -4.56]
Lin 2022	10.33	1.09	36	13.49	2.27	37	12.7%	-3.16 [-3.97, -2.35]
Qi 2024	3.57	0.93	30	3.63	0.72	30	13.4%	-0.06 [-0.48, 0.36]
Su 2024	7.27	2.96	49	11.46	4.13	49	11.0%	-4.19 [-5.61, -2.77]
Zhang 2023	7.3	4.2	50	4.4	3.9	48	10.5%	2.90 [1.30, 4.50]
**Subtotal (95% CI)**			**340**			**350**	**74.8%**	**-2.06 [-3.90, -0.21]**
Heterogeneity: $Tau^2 = 5.83$; $Chi^2 = 111.41$, df = 7 (P < 0.00001); $I^2 = 94\%$								
Test for overall effect: Z = 2.19 (P = 0.03)								
**Total (95% CI)**			**446**			**454**	**100.0%**	**-1.80 [-2.87, -0.72]**
Heterogeneity: $Tau^2 = 2.18$; $Chi^2 = 128.74$, df = 10 (P < 0.00001); $I^2 = 92\%$								
Test for overall effect: Z = 3.29 (P = 0.001)								
Test for subgroup differences: $Chi^2 = 0.36$, df = 1 (P = 0.55), $I^2 = 0\%$								

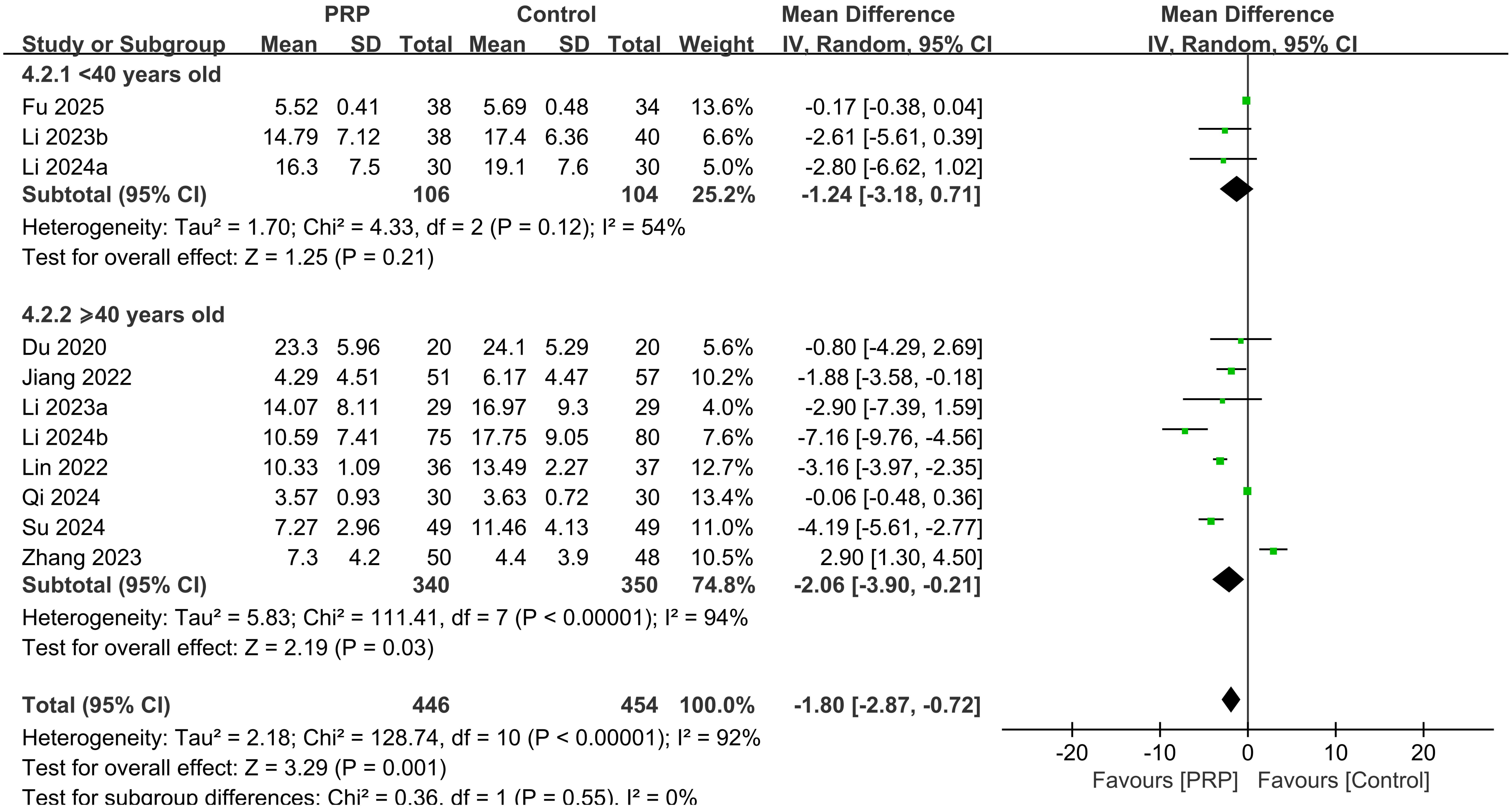

Fig. S8. Subgroup analysis of ODI scores by patient age.

Table S1

Sensitivity analysis for back VAS scores.

Eliminated study	Heterogeneity		Effect Model	MD	95% CI	P Value
	P Value	I^2 (%)				
None	0.001	64	Random	-0.23	-0.36 to -0.10	0.0005
Fu et al., 2025 [25]	0.001	56	Random	-0.26	-0.40 to -0.11	0.0004
Li et al., 2024 [26]	0.0008	67	Random	-0.23	-0.37 to -0.09	0.0010
Li et al., 2024 [27]	0.001	66	Random	-0.22	-0.37 to -0.08	0.002
Qi et al., 2024 [28]	0.001	65	Random	-0.25	-0.39 to -0.12	0.0003
Su 2024 [29]	0.11	36	Fix	-0.12	-0.19 to -0.05	0.0004
Li et al., 2023 [30]	0.0008	67	Random	-0.23	-0.37 to -0.09	0.001
Li et al., 2023 [31]	0.0008	67	Random	-0.23	-0.37 to -0.09	0.001
Zhang et al., 2023 [2]	0.001	56	Random	-0.19	-0.31 to -0.08	0.001
Lin et al., 2022 [32]	0.001	66	Random	-0.25	-0.39 to -0.11	0.0006
Jiang et al., 2022 [33]	0.001	66	Random	-0.22	-0.36 to -0.08	0.002
Lu et al., 2021 [34]	0.0008	67	Random	-0.24	-0.38 to -0.10	0.0007
Du et al., 2020 [35]	0.0008	67	Random	-0.23	-0.36 to -0.09	0.0009

Table S2

Sensitivity analysis leg VAS scores.

Eliminated study	Heterogeneity		Effect Model	MD	95% CI	P Value
	P Value	I^2 (%)				
None	0.006	70	Random	-0.35	-0.60 to -0.10	0.007
Li et al., 2024 [26]	0.004	74	Random	-0.37	-0.65 to -0.08	0.01
Li et al., 2024 [27]	0.002	76	Random	-0.33	-0.65 to -0.02	0.04
Su 2024 [29]	0.34	12	Fix	-0.26	-0.41 to -0.10	0.001
Li et al., 2023 [30]	0.003	76	Random	-0.34	-0.64 to -0.04	0.02
Zhang et al., 2023 [2]	0.06	56	Random	-0.44	-0.66 to -0.21	0.0001
Jiang et al., 2022 [33]	0.003	76	Random	-0.34	-0.65 to -0.03	0.03

Table S3

Sensitivity analysis for JOA scores.

Eliminated study	Heterogeneity		Effect Model	MD	95% CI	P Value
	P Value	I^2 (%)				
None	<0.00001	88	Random	1.26	0.31 to 2.21	0.009
Fu et al., 2025 [25]	<0.00001	90	Random	1.39	0.28 to 2.49	0.01
Li et al., 2024 [26]	<0.00001	90	Random	1.33	0.26 to 2.40	0.02
Li et al., 2024 [27]	<0.00001	90	Random	1.30	0.17 to 2.43	0.02
Li et al., 2023 [30]	<0.00001	90	Random	1.34	0.25 to 2.42	0.02
Li et al., 2023 [31]	<0.00001	90	Random	1.35	0.23 to 2.47	0.02
Zhang et al., 2023 [2]	0.64	0	Fix	0.64	0.31 to 0.97	0.0001
Lu et al., 2021 [34]	<0.00001	89	Random	1.44	0.37 to 2.52	0.009
Du et al., 2020 [35]	<0.00001	90	Random	1.27	0.23 to 2.31	0.02

Table S4

Sensitivity analysis for ODI scores.

Eliminated study	Heterogeneity		Effect Model	MD	95% CI	P Value
	P Value	I^2 (%)				
None	<0.00001	92	Random	-1.53	-2.45 to -0.61	0.001
Fu et al., 2025 [25]	<0.00001	92	Random	-1.85	-3.17 to -0.54	0.006
Li et al., 2024 [26]	<0.00001	92	Random	-1.47	-2.41 to -0.53	0.002
Li et al., 2024 [27]	<0.00001	90	Random	-1.12	-1.99 to -0.25	0.01
Qi et al., 2024 [28]	<0.00001	92	Random	-1.83	-3.05 to -0.62	0.003
Su 2024 [29]	<0.00001	90	Random	-1.18	-2.08 to -0.29	0.009
Li et al., 2023 [30]	<0.00001	92	Random	-1.48	-2.42 to -0.55	0.002
Li et al., 2023 [31]	<0.00001	92	Random	-1.46	-2.41 to -0.52	0.002
Zhang et al., 2023 [2]	<0.00001	91	Random	-1.95	-2.88 to -1.02	<0.0001
Lin et al., 2022 [32]	<0.00001	88	Random	-1.20	-2.06 to -0.35	0.006
Jiang et al., 2022 [33]	<0.00001	92	Random	-1.49	-2.46 to -0.53	0.002
Lu et al., 2021 [34]	<0.00001	92	Random	-1.80	-2.87 to -0.72	0.001
Du et al., 2020 [35]	<0.00001	92	Random	-1.56	-2.51 to -0.62	0.001

Table S5

Sensitivity analysis for disc height.

Eliminated study	Heterogeneity		Effect Model	MD	95% CI	P Value
	P Value	I^2 (%)				
None	0.61	0	Fix	0.74	0.52 to 0.97	<0.00001
Fu et al., 2025 [25]	0.47	0	Fix	0.75	0.52 to 0.99	<0.00001
Su 2024 [29]	0.53	0	Fix	0.76	0.53 to 0.99	<0.00001
Li et al., 2023 [30]	0.71	0	Fix	0.81	0.56 to 1.06	<0.00001
Zhang et al., 2023 [2]	0.87	0	Fix	0.58	0.26 to 0.89	0.0003
Jiang et al., 2022 [33]	0.57	0	Fix	0.76	0.53 to 0.99	<0.00001
Du et al., 2020 [35]	0.46	0	Fix	0.74	0.48 to 0.99	<0.00001

Table S6

Sensitivity analysis for complication.

Eliminated study	Heterogeneity		Effect Model	RR	95% CI	P Value
	P Value	I^2 (%)				
None	0.48	0	Fix	0.81	0.38 to 1.73	0.58
Fu et al., 2025 [25]	0.53	0	Fix	1.21	0.42 to 3.51	0.73
Lin et al., 2022 [32]	0.30	8	Fix	0.67	0.24 to 1.86	0.44
Du et al., 2020 [35]	0.40	0	Fix	0.72	0.32 to 1.59	0.42

Table S7

Sensitivity analysis for recurrence.

Eliminated study	Heterogeneity		Effect Model	RR	95% CI	P Value
	P Value	I^2 (%)				
None	0.99	0	Fix	0.27	0.12 to 0.60	0.001
Li et al., 2024 [26]	0.98	0	Fix	0.26	0.11 to 0.62	0.002
Li et al., 2024 [27]	1.00	0	Fix	0.31	0.13 to 0.74	0.009
Li et al., 2023 [30]	0.98	0	Fix	0.26	0.11 to 0.62	0.002
Li et al., 2023 [31]	0.98	0	Fix	0.27	0.11 to 0.64	0.003
Zhang et al., 2023 [2]	0.99	0	Fix	0.25	0.10 to 0.65	0.005
Jiang et al., 2022 [33]	0.98	0	Fix	0.26	0.11 to 0.64	0.003

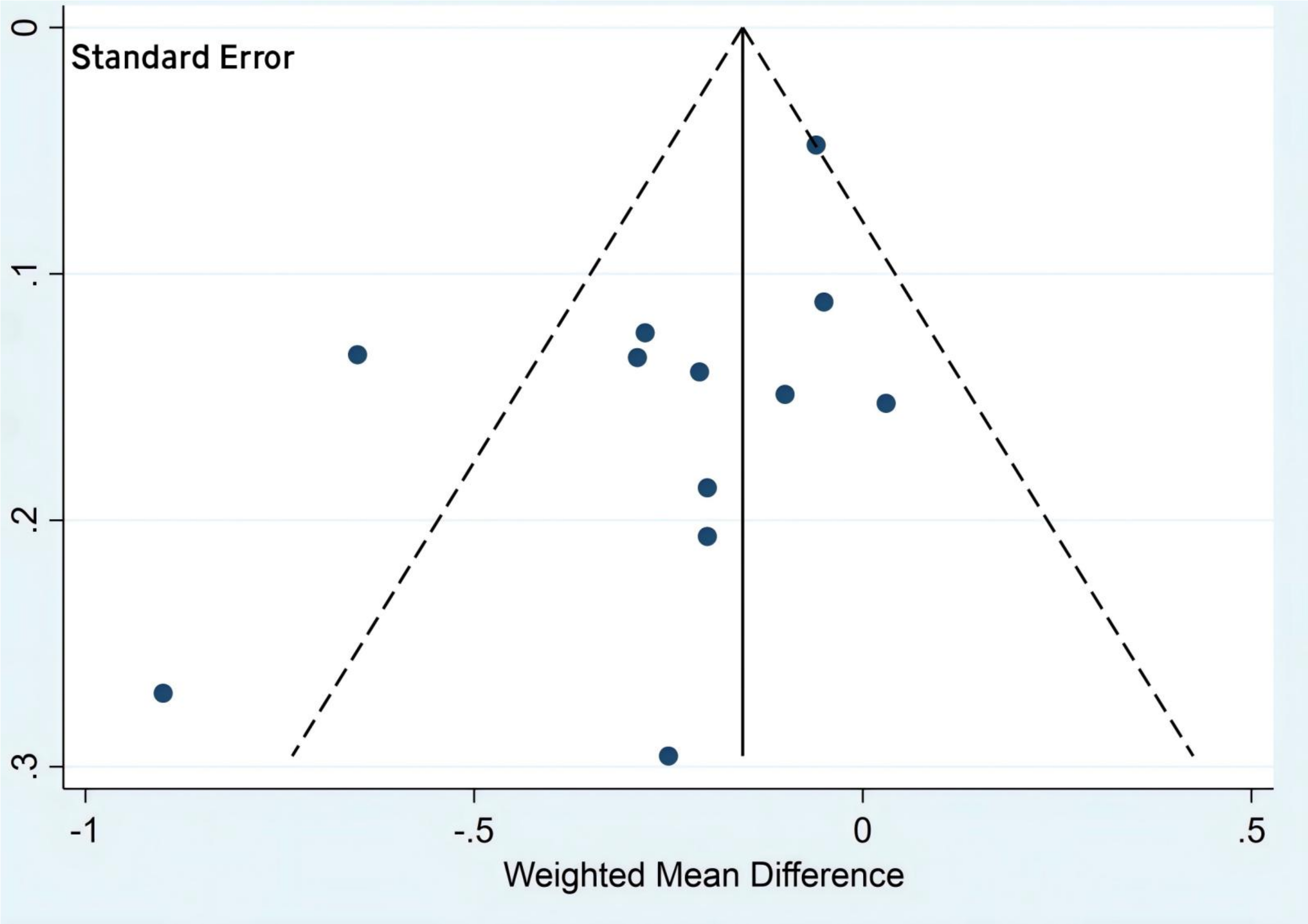

Fig. S9. The funnel plot of back VAS scores. Each point represents an individual study.

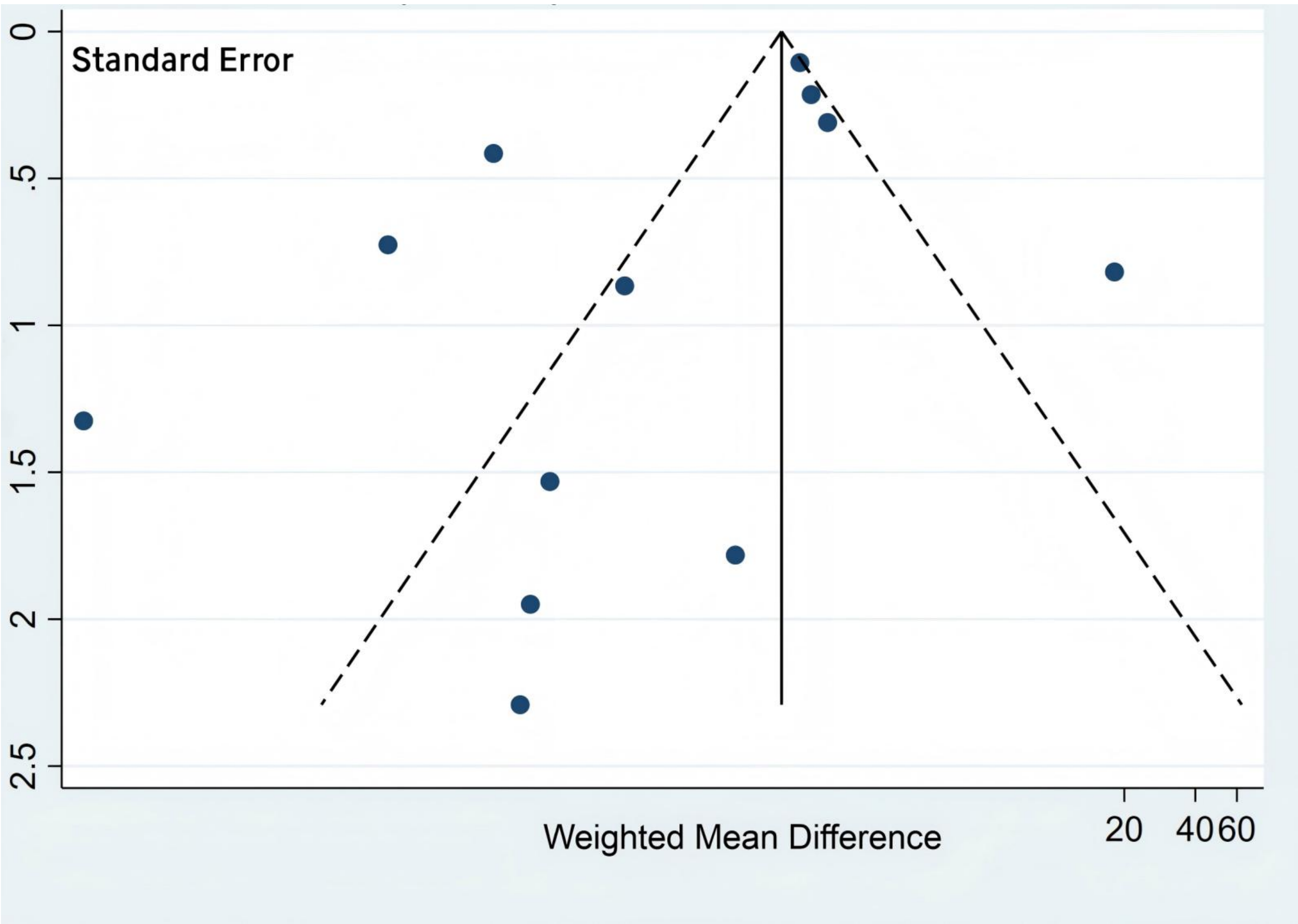

Fig. S10. The funnel plot of ODI scores. Each point represents an individual study.

Table S8

Publication bias of the included studies.

Outcomes	Number of trails	Egger's test (P value)
Back VAS scores	12 studies [2,25-35]	0.067
ODI scores	12 studies [2,25-35]	0.105

Table S9

GRADE evaluation of evidence quality.

Outcome	Point estimate (95% CI)	Trials (participants)	GRADE (assessment by the review)	Comments
Back VAS scores	-0.23 [-0.36 to -0.10]	12 studies (960)	Very low	Study design: Mixture of RCTs and observational studies (Initial level: Low)<br>Risk of bias: No studies at high RoB<br>Inconsistency: I^2 = 64% (downgraded)<br>Indirectness: Not downgraded<br>Imprecision: >100 participants<br>Publication bias: No evidence
Leg VAS scores	-0.35 [-0.60 to -0.10]	6 studies (577)	Very low	Study design: Mixture of RCTs and observational studies (Initial level: Low)<br>Risk of bias: No studies at high RoB<br>Inconsistency: I^2 = 70% (downgraded)<br>Indirectness: Not downgraded<br>Imprecision: >100 participants<br>Publication bias: No evidence
JOA scores	1.26 [0.31 to 2.21]	8 studies (621)	Very low	Study design: Mixture of RCTs and observational studies (Initial level: Low)<br>Risk of bias: No studies at high RoB<br>Inconsistency: I^2 = 88% (downgraded)<br>Indirectness: Not downgraded<br>Imprecision: >100 participants<br>Publication bias: No evidence
ODI scores	-1.53 [-2.45 to -0.61]	12 studies (960)	Very low	Study design: Mixture of RCTs and observational studies (Initial level: Low)<br>Risk of bias: No studies at high RoB<br>Inconsistency: I^2 = 92% (downgraded)<br>Indirectness: Not downgraded

				Imprecision: >100 participants<br>Publication bias: No evidence
Disc height	0.74 [0.52 to 0.97]	6 studies (474)	Low	Study design: Mixture of RCTs and observational studies (Initial level: Low)<br>Risk of bias: No studies at high RoB<br>Inconsistency: $I^2 = 0\%$<br>Indirectness: Not downgraded<br>Imprecision: >100 participants<br>Publication bias: No evidence
Complications	0.81 [0.38 to 1.73]	3 studies (185)	Very low	Study design: Mixture of RCTs and observational studies (Initial level: Low)<br>Risk of bias: No studies at high RoB<br>Inconsistency: $I^2 = 0\%$<br>Indirectness: Not downgraded<br>Imprecision: Total number of participants <100 events (downgraded)<br>Publication bias: No evidence
Recurrence	0.27 [0.12 to 0.60]	6 studies (557)	Low	Study design: Observational studies (Initial level: Low)<br>Risk of bias: No studies at high RoB<br>Inconsistency: $I^2 = 0\%$<br>Indirectness: Not downgraded<br>Imprecision: Total number of participants >100 events<br>Publication bias: No evidence
